# Molecularly defined circuits for cardiovascular and cardiopulmonary control

**DOI:** 10.1038/s41586-022-04760-8

**Published:** 2022-06-01

**Authors:** Avin Veerakumar, Andrea R. Yung, Yin Liu, Mark A. Krasnow

**Affiliations:** 1 Department of Biochemistry and Howard Hughes Medical Institute, Stanford University School of Medicine, Stanford, CA USA;; 2 Department of Bioengineering, Stanford University, Stanford, CA USA;; 3 Medical Scientist Training Program, Stanford University School of Medicine, Stanford, CA USA

## Abstract

The sympathetic and parasympathetic nervous systems powerfully regulate internal organs^1^, but the molecular and functional diversity of their constituent neurons and circuits remains largely unknown. Here we use retrograde neuronal tracing, single-cell RNA sequencing, optogenetics, and physiological experiments to dissect the cardiac parasympathetic control circuit in mice. We show that cardiac-innervating neurons in the brainstem nucleus ambiguus (Amb) are comprised of two molecularly, anatomically, and functionally distinct subtypes. One we call ACV (ambiguus cardiovascular) neurons (~35 neurons per Amb), define the classical cardiac parasympathetic circuit. They selectively innervate a subset of cardiac parasympathetic ganglion neurons and mediate the baroreceptor reflex, slowing heart rate and atrioventricular node conduction in response to increased blood pressure. The other, ACP (ambiguus cardiopulmonary) neurons (~15 neurons per Amb) innervate cardiac ganglion neurons intermingled with and functionally indistinguishable from those innervated by ACV neurons, but surprisingly also innervate most or all lung parasympathetic ganglion neurons; clonal labeling shows individual ACP neurons innervate both organs. ACP neurons mediate the dive reflex, the simultaneous bradycardia and bronchoconstriction that follows water immersion. Thus, parasympathetic control of the heart is organized into two parallel circuits, one that selectively controls cardiac function (ACV circuit) and another that coordinates cardiac and pulmonary function (ACP circuit). This new understanding of cardiac control has implications for treating cardiac and pulmonary diseases and for elucidating the control and coordination circuits of other organs.

## Introduction

The autonomic nervous system is a vast network of neurons under involuntary control that is essential for maintaining physiological homeostasis throughout the body. In 1921, Langley divided the autonomic nervous system into the opposing sympathetic and parasympathetic nervous systems, which generally act antagonistically within a given organ through release of norepinephrine (typically mediating “fight or flight” responses) and acetylcholine (typically mediating “rest and digest” responses), respectively^1^. This provided a foundation for modern physiology and medicine that has remained mostly intact a century later^2^. Despite the vaunted position of the parasympathetic and sympathetic nervous systems, the molecular, cellular, and functional diversity of their constituent neurons and circuits remains largely unknown, most notably at the central nervous system level^2,3^.

One critical organ controlled by the autonomic nervous system is the heart. The parasympathetic and sympathetic nervous systems powerfully influence heart rate, rhythm, and contractility to meet internal and external demands^4^. Classical physiological studies have shown that the sympathetic nervous system exerts control of heart rate on a time scale of seconds, while the parasympathetic nervous system exerts control on an even faster, beat-to-beat timescale and is thus thought to be responsible for the tight coordination between cardiac and pulmonary function^5^. Cardiac parasympathetic outflow originates from brainstem preganglionic neurons, residing primarily in the nucleus ambiguus (Amb) in the medulla^4^. A minority of cardiac parasympathetic neurons are located in the dorsal motor nucleus of vagus, which controls ventricular inotropy and excitability, but does not control heart rate^6^. Amb neurons innervate cholinergic neurons in parasympathetic cardiac ganglia located on the surface of the heart, and these ganglion neurons in turn innervate the sinoatrial (SA) node, atrioventricular (AV) node, and myocardial tissue. Acetylcholine release from these ganglion neurons reduces heart rate, AV conduction velocity, and myocardial contractility by engaging cardiac muscarinic receptors. Altered sympathetic-parasympathetic signaling balance to the heart has been implicated in a wide range of cardiovascular diseases^7–9^, and is molecularly targeted by common cardiac therapies such as β-blockers and atropine, but the neuronal cell types within the cardiac parasympathetic and sympathetic nervous systems have not been molecularly defined.

## Results

### Targeted scRNAseq of Amb^Cardiac^ neurons

**Extended Data Figure 1.**
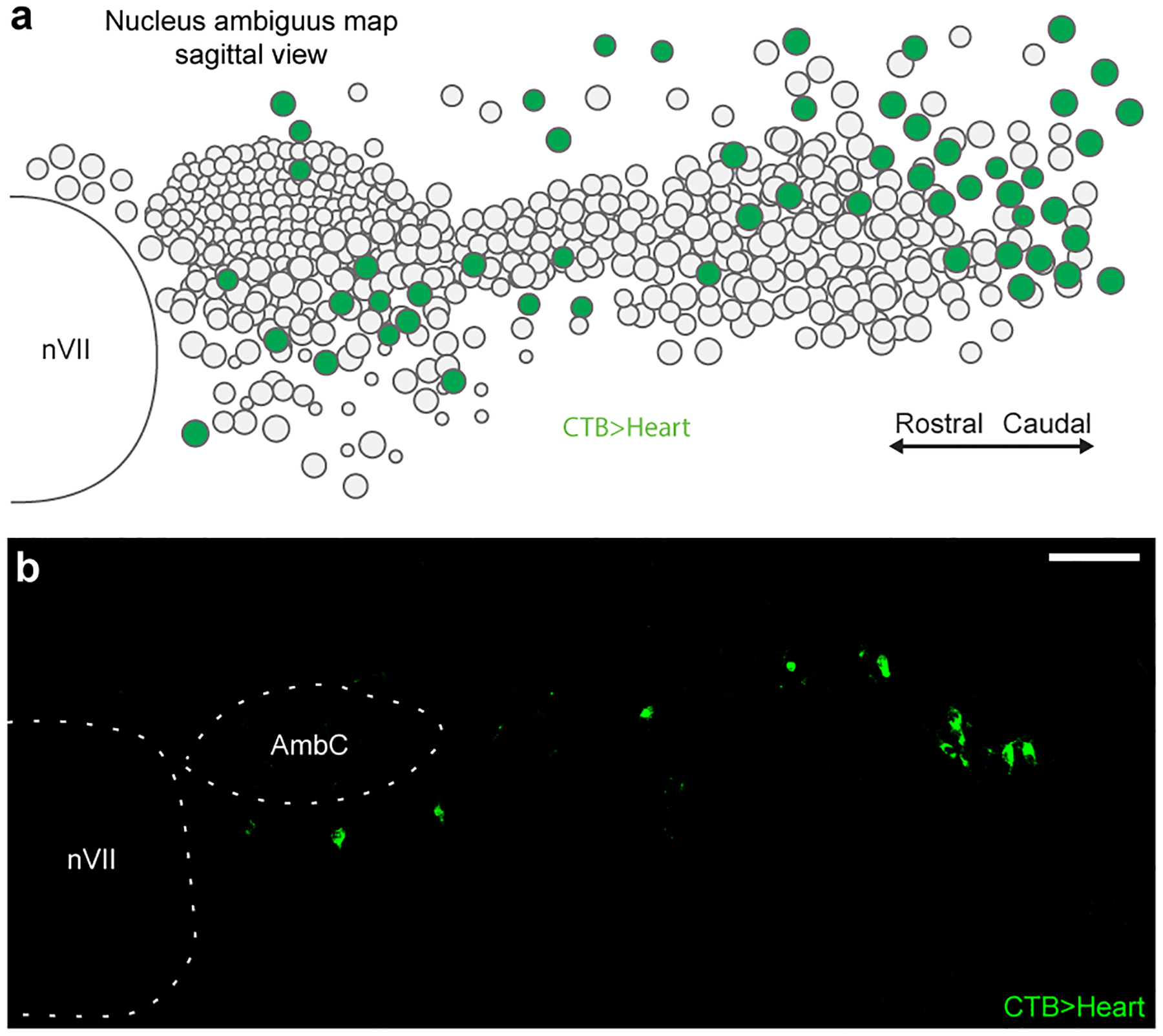
Distribution of cardiac-innervating neurons in the nucleus ambiguus (Amb). **a**, Sagittal schematic view displaying locations of Amb neurons (circles) within a postnatal day 2 Amb nucleus. Map is an overlay showing the locations of Amb neurons from all sagittal sections spanning a single Amb nucleus. Green fill circles, cardiac Amb neurons retrograde labeled by cholera toxin B (CTB) injection into pericardial space (CTB>Heart). Grey fill circles, other Amb neurons. Note cardiac Amb neurons localize primarily to “external formation” of Amb, surrounding the principal rostral-caudal column of Amb neurons. **b**, Representative sagittal section of Amb with Amb^Cardiac^ neurons labeled in green by retrograde labeling by CTB injection in heart (CTB>Heart). Retrograde labeled cells localized to the Amb external formation. AmbC, nucleus ambiguus compact formation. nVII, facial motor nucleus. Bar, 100 μm.

**Extended Data Figure 2.**
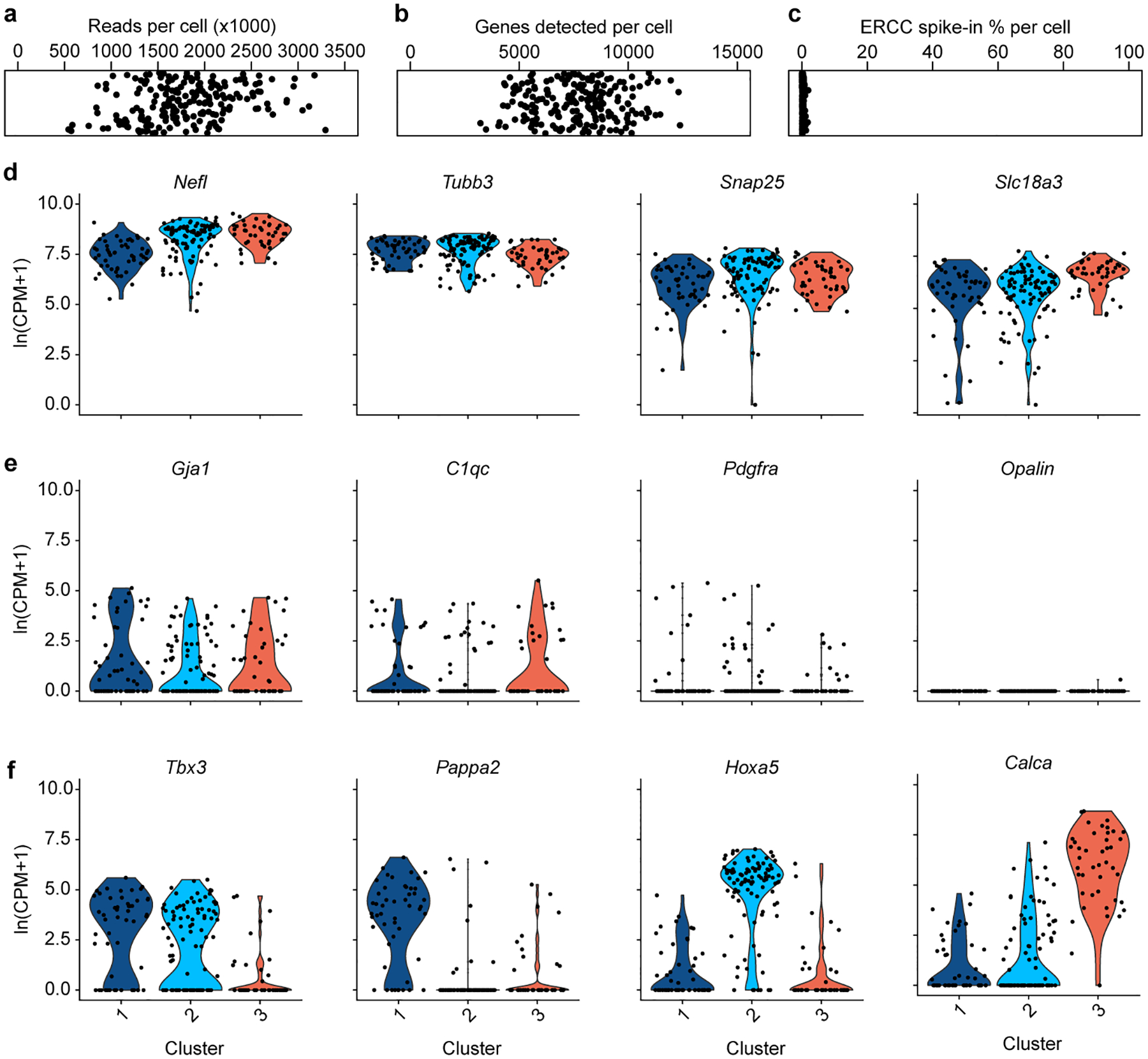
Quality control data for Amb single cell RNAseq. **a**, Aligned reads per cell in combined Amb^Cardiac^ and Amb^Laryngeal^ dataset. Cells with less than 500,000 reads were excluded from analysis. **b**, Genes detected per cell. **c**, Percent of reads per cell that aligned to ERCC spike-in RNA. **d**, Pan-neuronal genes *Nefl*, *Tubb3*, *Snap25*, and cholinergic gene *Slc18a3* were highly expressed across all clusters from Fig. 1b. **e**, Glial genes were expressed at similarly low levels across the 3 clusters from Fig. 1b, indicating they did not contribute to clustering. *Gja1*; astrocyte marker, *C1qc*; microglia marker, *Pdgfra*; oligodendrocyte precursor cell marker, *Opalin*; oligodendrocyte marker. **f**, Examples of marker genes that significantly contributed to clustering of the 3 neuron types (*Tbx3*; Amb^Cardiac^ marker, *Pappa2*; ACP marker, *Hoxa5*; ACV marker, *Calca*; Amb^Laryngeal^ marker).

**Figure 1.**
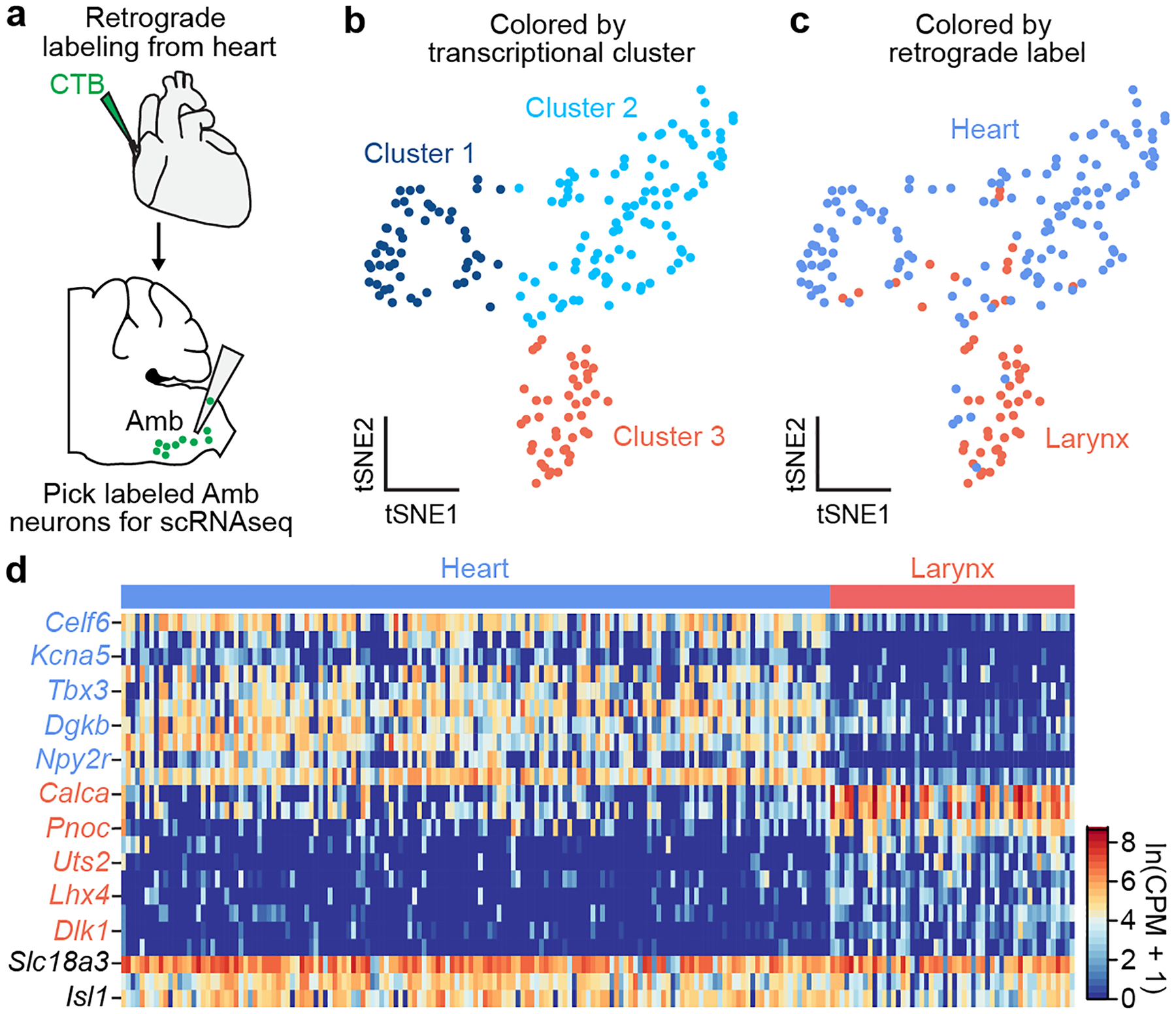
scRNAseq of Amb^Cardiac^ neurons identifies a genetic signature of brainstem parasympathetic neurons. **a**, Strategy for isolating Amb^Cardiac^ neurons. Fluorescent CTB was injected into the pericardial space, and 1–3 days later the retrograde-labeled cardiac-innervating neurons in the nucleus ambiguus (Amb^Cardiac^ neurons) were aspirated from acute physiological brainstem slices, then processed for scRNAseq. In separate mice (not shown), CTB was injected into the cricothyroid laryngeal muscle to similarly visualize, aspirate, and process control Amb^Laryngeal^ neurons. **b**, t-distributed stochastic neighbor embedding (tSNE) plot comparing Amb neuron scRNA-seq expression profiles (dots). Note three transcriptionally distinct neuronal clusters defined by nearest neighbor analysis. **c**, tSNE plot of same Amb neurons as (b) but colored by their retrograde label origin. Amb^Laryngeal^ neurons largely comprise cluster 3, whereas Amb^Cardiac^ neurons were largely split between clusters 1 and 2. **d**, Heat map showing log-transformed expression levels of selected genes differentially expressed between Amb^Cardiac^ and Amb^Laryngeal^ neurons. Names in black, expected pan-Amb genes. Mouse brain image and those in Figs. 3, 4, 6, 7 and Extended Data Figs. 5 and 7 are reproduced from the Paxinos and Franklin atlas^35^.

To molecularly define parasympathetic neurons that control the heart, we retrogradely labeled and transcriptionally profiled cardiac-innervating neurons from the mouse brainstem using single-cell RNA sequencing (scRNAseq). Wild-type postnatal day 1 mice were injected with fluorescent retrograde neuronal tracer cholera toxin B into the pericardial space to label parasympathetic ganglia across the epicardial surface. After one day to allow tracer uptake into pre-ganglionic terminals and retrograde transport to the cell bodies in the brainstem, ~50–60 fluorescently-labeled cell bodies were observed in the nucleus ambiguus (Amb). Labeled cells (hereafter called Amb^Cardiac^ neurons) were distributed bilaterally in Amb in a stereotyped pattern (Extended Data Fig. 1); similar distributions of Amb^Cardiac^ neurons have been observed in adult rats^10^, cats^11^, and dogs^12^. Because of the small number of Amb^Cardiac^ neurons, we aspirated whole retrograde labeled Amb neurons directly from acute brainstem slices (Fig. 1a). We did the same for an intermingled outgroup population of Amb neurons that does not innervate the heart, by retrograde labeling and aspirating neurons that innervate the cricothyroid laryngeal muscle (hereafter called Amb^Laryngeal^ neurons, ~28 per Amb). Following sequencing, 151 Amb^Cardiac^ neurons and 52 Amb^Laryngeal^ neurons passed quality control (mean: 1.9 million reads, 7761 genes detected per cell, Extended Data Fig. 2), suggesting we achieved cellular saturation by sequencing more than the numbers of Amb^Cardiac^ and Amb^Laryngeal^ neurons per Amb.

Graph-based clustering of the expression profiles of the combined Amb^Cardiac^ and Amb^Laryngeal^ neuron datasets revealed three clusters of cells (Fig. 1b). One (Cluster 3) largely corresponded to Amb^Laryngeal^ neurons (Fig. 1c) and the two others to Amb^Cardiac^ neurons. Comparison of Amb^Cardiac^ and Amb^Laryngeal^ expression profiles identified over 500 differentially-expressed genes (Supplementary Tables 1, 2), including two known markers of Amb^Laryngeal^ neurons, neuropeptide genes *Calca* and *Calcb*^13^, as well as *Dlk1*, a regulator of somatic fast motor neuron fate^14^ (Fig. 1d). The ~390 genes selectively expressed in Amb^Cardiac^ neurons (Supplementary Tables 2, 3) include transcriptional (*Tbx3*) and splicing (*Celf6*) regulators, as well as genes that regulate synaptic connectivity or projection targets (*Cntn5*, *Cdh8*, *Efna5*), electrophysiological properties (*Kcna5*), input signaling (*Npy2r* and *Cnr1*), and disease processes (*Snca*).

**Extended Data Figure 3.**
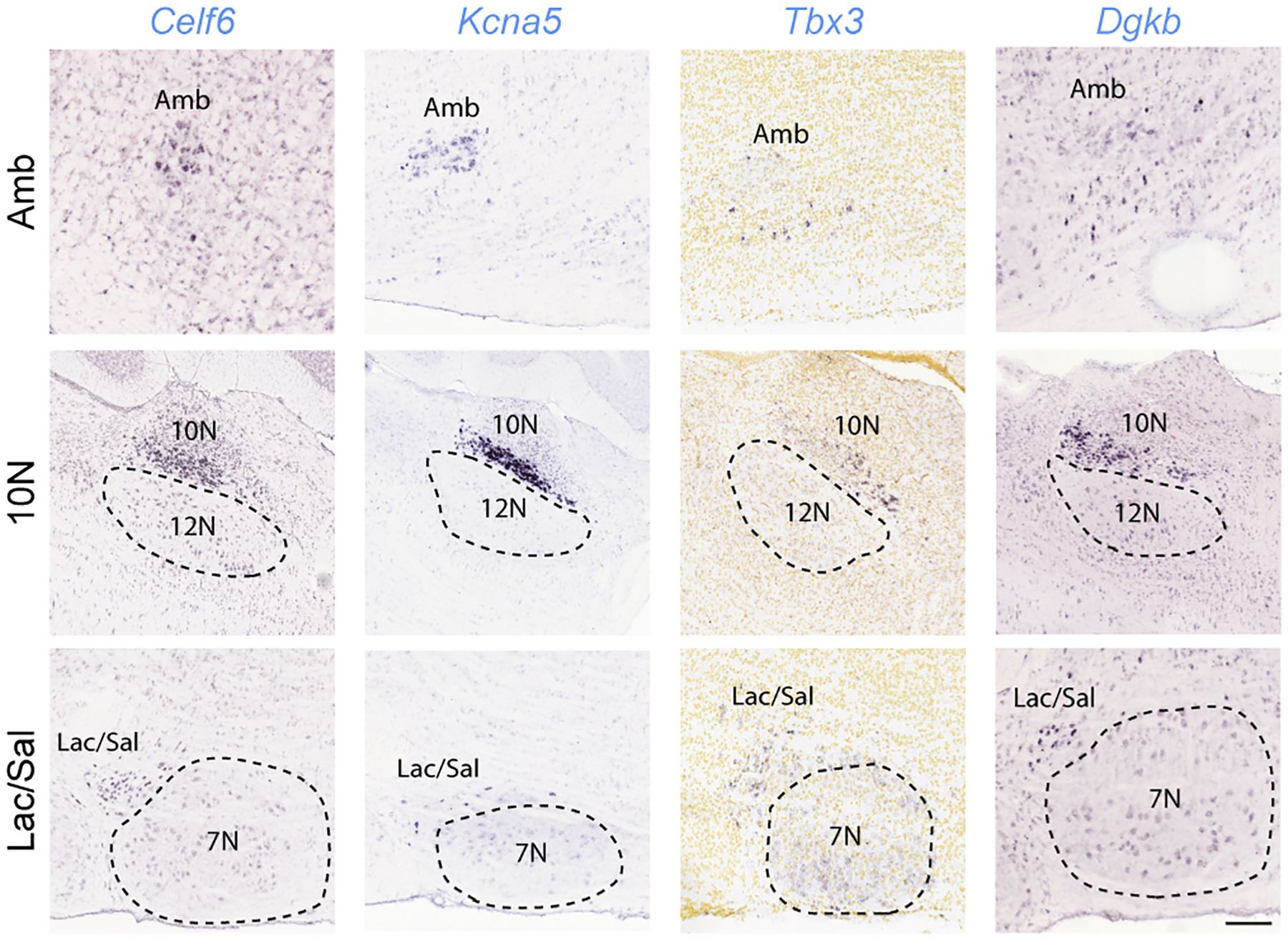
Amb^Cardiac^ markers are expressed in other brainstem parasympathetic nuclei. In situ hybridization data (from Allen Brain Atlas^15^) showing expression (dark purple) of the indicated Amb^Cardiac^-specific genes (*Celf6*, *Kcna5*, *Tbx3*, *Dgkb*, from Fig. 1) in parasympathetic neurons across the brainstem. Top row, Amb; middle row, dorsal motor nucleus of the vagus (10N); bottom row, lacrimal and salivatory nuclei (Lac/Sal). Note the genes are expressed in a subset of Amb neurons (top row panels), as predicted by scRNAseq (Fig. 1), but also in parasympathetic neurons of the dorsal motor nucleus of vagus (10N) that innervate thoracic and abdominal viscera (second row panels). Note absence of expression in the adjacent somatic motor neurons of the hypoglossal nucleus (12N). All four genes are also expressed in parasympathetic neurons of the facial nerve controlling the lacrimal and salivary glands (Lac/Sal) (third row panels) but had absent or lower expression in the adjacent facial motor nucleus (7N). Allen Brain Atlas images obtained from available postnatal day 56 sections (*Celf6*, *Kcna5*, *Dgkb*) and embryonic day 18.5 sections (*Tbx3*). Amb sections are sagittal except for *Celf6* (coronal). 10N and Lac/Sal sections are sagittal. Bar of 200 μm applies to all sections.

To determine if the ascertained Amb^Cardiac^ genetic signature includes genes that more generally define brainstem parasympathetic neurons, we used the Allen Brain Atlas^15^ to examine expression of available Amb^Cardiac^-specific genes in other brainstem parasympathetic nuclei. This identified 26 Amb^Cardiac^-specific genes including *Celf6*, *Kcna5*, *Tbx3* and *Dgkb* that are also expressed in parasympathetic neurons of the dorsal motor nucleus of vagus (10N), which controls thoracic and abdominal viscera, as well as parasympathetic neurons of the facial nerve controlling the lacrimal and salivary glands (Lac/Sal) (Extended Data Fig. 3, Supplementary Table 3). These genes are not expressed in adjacent somatic cranial motor nuclei, suggesting they are general markers of visceromotor neurons; we confirmed their expression in a recent adult scRNAseq dataset from 10N^3^. We also found Amb^Cardiac^-specific genes that were expressed in 10N but not Lac/Sal or were not expressed in brainstem parasympathetic nuclei other than Amb (Supplementary Table 3). Thus, our scRNAseq analysis of Amb^Cardiac^ neurons identified both general features of brainstem parasympathetic neurons and specific features of Amb^Cardiac^ neurons.

### Two types of Amb^Cardiac^ neurons

**Figure 2.**
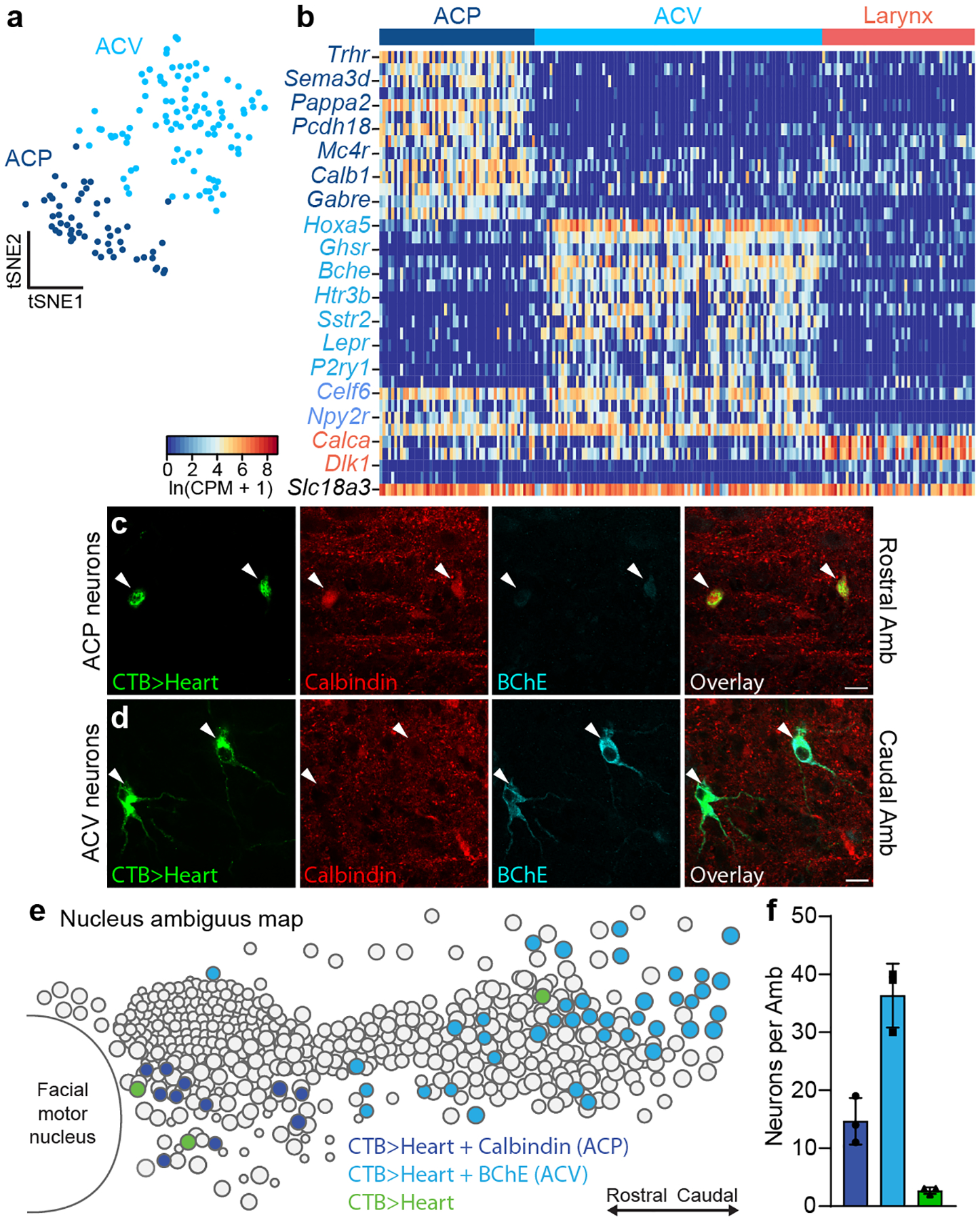
Two molecularly and anatomically distinct types of Amb^Cardiac^ neurons. **a**, tSNE plot of Amb^Cardiac^ neuron expression profiles (dots). Nearest neighbor analysis defined two molecularly distinct clusters designated ACP (dark blue) and ACV (light blue) for reasons described later. **b**, Heat map showing expression of selected genes enriched in ACP neurons (dark blue gene names), ACV neurons (light blue), all Amb^Cardiac^ neurons (purple), all Amb^Laryngeal^ neurons (“Larynx”, coral), and all Amb neurons (black). **c**, Immunostaining for ACP marker Calbindin and ACV marker BChE in rostral Amb. Rostral Amb neurons (arrowheads) labeled by intrapericardial CTB stained positive for Calbindin but expressed low levels of BChE, indicating they were ACP neurons. **d**, Immunostaining for ACP marker Calbindin and ACV marker BChE in caudal Amb of same sample as panel c. Caudal Amb neurons (arrowheads) labeled by intrapericardial CTB expressed high levels of BChE but stained negative for Calbindin, indicating they were ACV neurons. Bars, 20 μm. **e**, Map of ACP and ACV neurons in Amb. Sagittal schematic view showing soma of all neurons (circles) in a representative single postnatal day 2 Amb that was retrograde labeled with CTB from the heart (CTB>Heart) and stained for ACP marker Calbindin (dark blue circles) and ACV marker butyrylcholinesterase (BChE, light blue circles). A minority of retrograde-labeled cardiac neurons (green circles) did not stain for Calbindin or BChE. **f**, Quantification of absolute numbers of ACP neurons (dark blue), ACV neurons (light blue), and double negative cardiac neurons (green) per Amb (mean ± s.d., *n* = 3 mice).

Sub-clustering of Amb^Cardiac^ neurons (Fig. 1b,c) without Amb^Laryngeal^ neurons confirmed they are not a homogeneous population, and identified two molecular subtypes (Fig. 2a) that did not further sub-cluster. We designated these subtypes ACP and ACV, for reasons described later. Both ACP and ACV expressed similarly high levels of cholinergic marker *Slc18a3* and cranial motor neuron transcription factor *Isl1* (Fig. 2b), as expected for Amb neurons^16^, whereas over 200 genes distinguished the two molecular types (Supplementary Tables 4, 5, 6). The differentially-expressed genes included neuropeptide and neurotransmitter receptors such as thyrotropin-releasing hormone receptor (*Trhr*), tachykinin receptor 3 (*Tacr3*), and melanocortin receptor 4 (*Mc4r*) that were selectively expressed in the ACP population, as well as growth hormone secretagogue receptor (*Ghsr*), serotonin receptor 3B (*Htr3b*), somatostatin receptors 2 and 5 (*Sstr2*, *Sstr5*), purinergic receptor P2Y1 (*P2ry1*), and leptin receptor (*Lepr*) that were selectively expressed in ACV. We also found subtype-specific transcription factor genes, neuropeptide precursor genes, voltage-gated ion channel genes, and genes known to regulate axon guidance and neuronal connectivity (Supplementary Table 6).

**Extended Data Figure 4.**
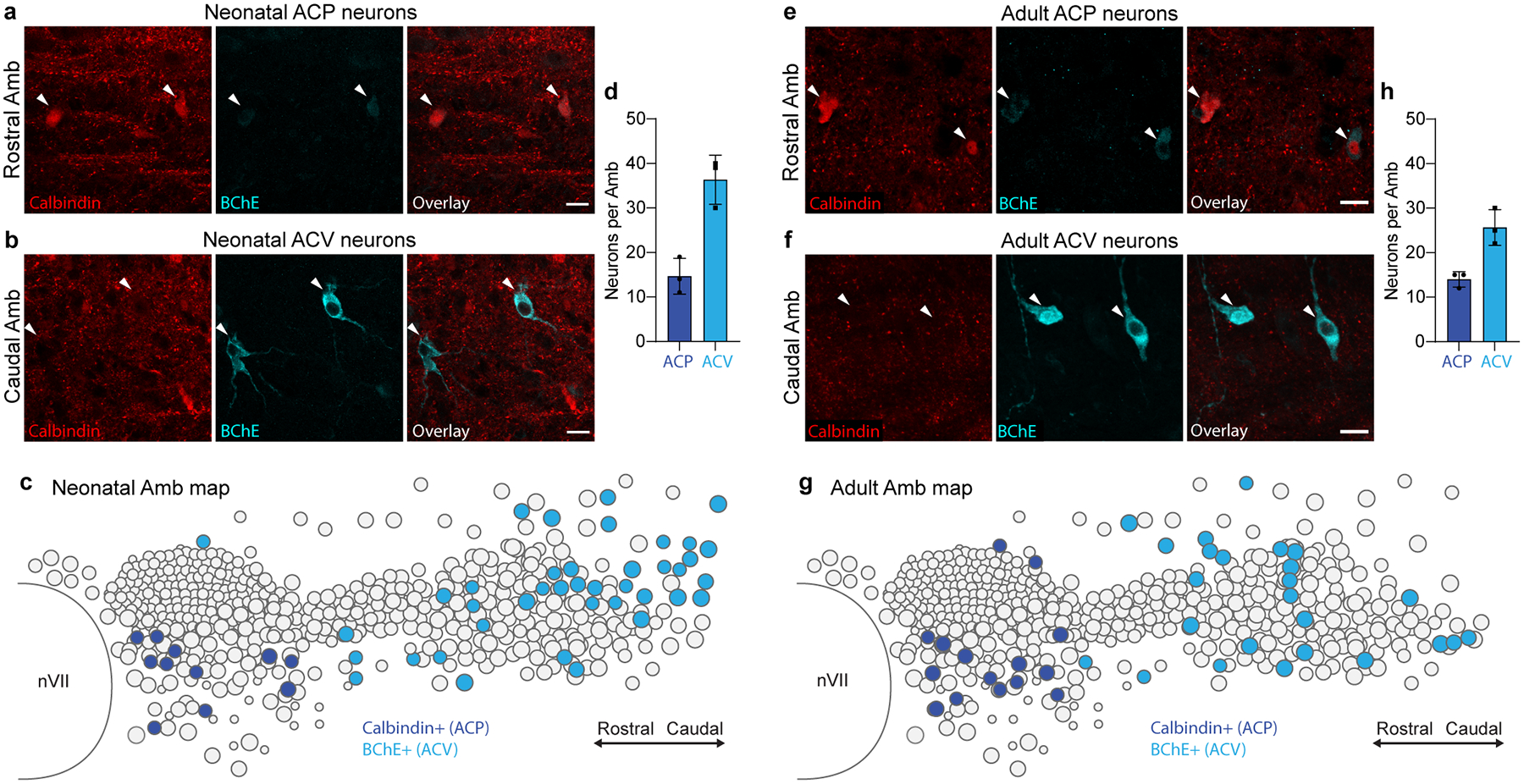
Conservation of ACP and ACV markers across postnatal development. **a**, Immunostaining of ACP neurons in the rostral Amb of a neonatal (postnatal day 2) mouse. ACP neurons stained positive for calbindin (red) and stained weakly positive for BChE (cyan). **b**, Immunostaining of neonatal ACV neurons in the caudal Amb of a postnatal day 2 mouse. ACV neurons stained positive for BChE and negative for calbindin. Bars, 20 μm. **c**, Map of ACP and ACV neurons in neonatal Amb. Sagittal schematic view showing overlay of soma of all neurons (circles) across all sections spanning a single neonatal (postnatal day 2) Amb that was stained for ACP marker calbindin (dark blue circles) and ACV marker BChE (light blue circles). **d**, Quantification of absolute numbers of ACP neurons (calbindin+, dark blue) and ACV neurons (BChE+, light blue) per Amb in neonatal mice (mean ± s.d., *n* = 3 mice). **e**, Immunostaining of ACP neurons in the rostral Amb of an adult (postnatal day 60) mouse. ACP neurons stained positive for calbindin (red) and stained weakly positive for BChE (cyan). **f**, Immunostaining of adult ACV neurons in the caudal Amb of a postnatal day 60 mouse. ACV neurons stained positive for BChE and negative for calbindin. Bars, 20 μm. **g**, Map of ACP and ACV neurons in adult Amb. Sagittal schematic view showing overlay of soma of all neurons (circles) across all sections spanning a single adult (postnatal day 60) Amb that was stained for calbindin (dark blue) and BChE (light blue). Note similarity in marker expression and cell type distribution in neonatal and adult Amb. **h**, Quantification of absolute numbers of ACP neurons (calbindin+, dark blue) and ACV neurons (BChE+, light blue) per Amb in adult mice (mean ± s.d., *n* = 3 mice).

To localize the two molecular types of Amb^Cardiac^ neurons in the brainstem, we retrogradely labeled Amb^Cardiac^ neurons and immunostained for ACP marker Calbindin (*Calb1*) and ACV marker Butyrylcholinesterase (*Bche*). As predicted by scRNAseq, Amb^Cardiac^ neurons consisted of one subpopulation that expressed high levels of Calbindin and low levels of BChE (ACP) (Fig. 2c), and a second subpopulation with high levels of BChE and undetectable Calbindin (ACV) (Fig. 2d), with only rare neurons (2–3 neurons per Amb) expressing neither marker. Strikingly, we observed a stereotyped topographic arrangement of the two subpopulations. ACP neurons (~10–20 per nucleus) were located in the rostral Amb, below the compact formation of the nucleus, whereas ACV neurons (~30–40 per nucleus) were located exclusively caudal to ACP, surrounding the loose formation of the nucleus (Fig. 2e,f). ACP and ACV marker expression and distributions were similar in adult mice (Extended Data Fig. 4, Supplementary Table 6). Thus, Amb^Cardiac^ neurons are comprised of two molecularly and anatomically distinct subtypes, ACP and ACV.

### ACP and ACV target distinct heart neurons

**Extended Data Figure 5.**
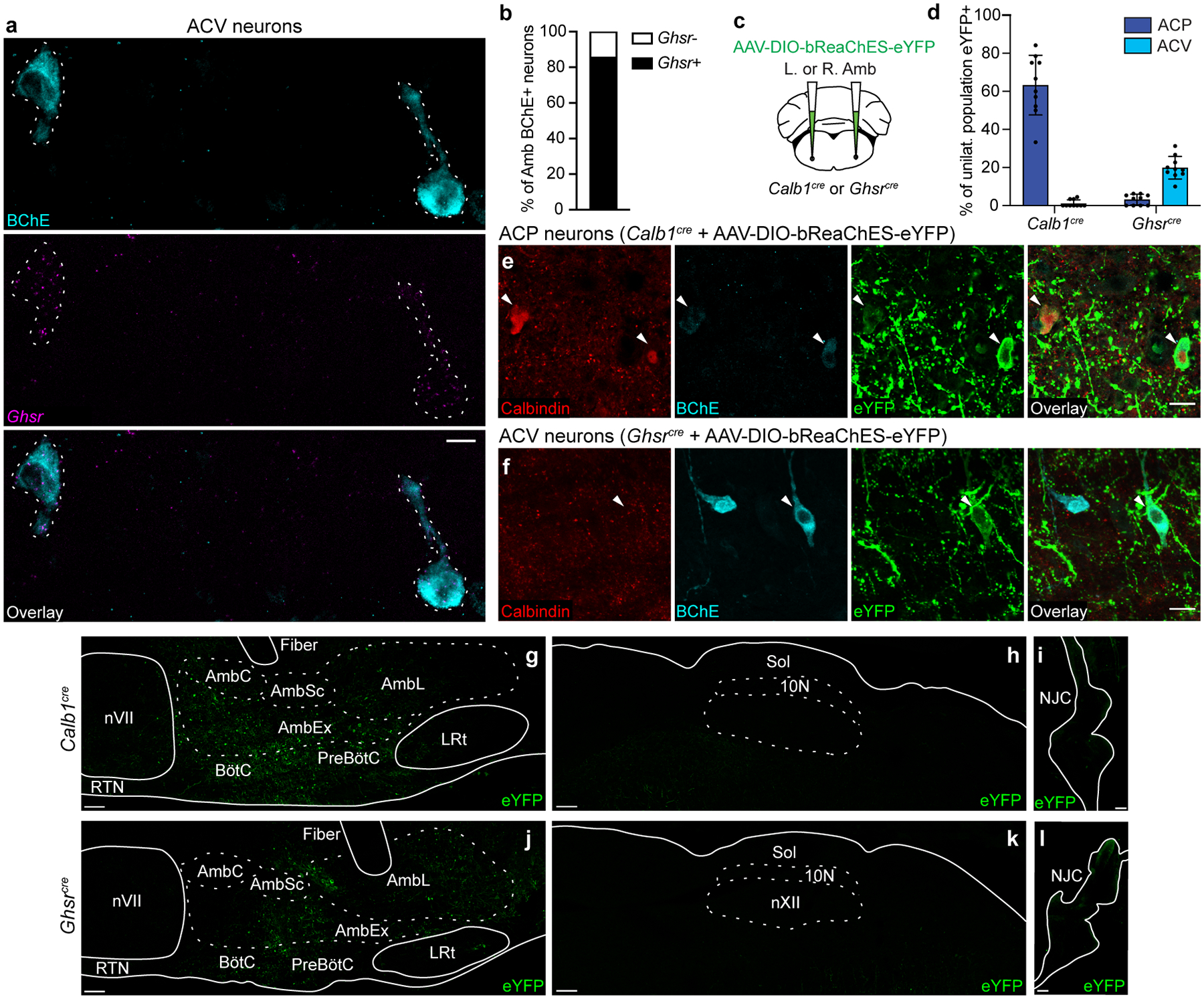
Viral targeting of ACP or ACV neurons. **a**, Combined immunostaining and smFISH showing overlap between BChE protein expression (cyan) and *Ghsr* mRNA expression (purple) in ACV neurons (dashed outlines). Bar, 100 μm. **b**, Quantification of panel a showing fraction of BChE+ ACV neurons that express *Ghsr* in Amb (*n* = 3 mice, 21 neurons total). **c**, Strategy for targeting ACP or ACV neurons. A Cre-dependent AAV encoding the opsin bReaChES fused to eYFP was delivered to the left or right Amb in *Calb1*^*cre*^ mice (to target ACP neurons) or *Ghsr*^*cre*^ mice (to target ACV neurons). **d**, Targeting specificity of AAV-DIO-bReaChES-eYFP vector used for left- and right-sided terminal mapping and optogenetics experiments in Figs. 3–4 (*n* = 10 *Calb1*^*cre*^, *n* = 10 *Ghsr*^*cre*^ mice, *n* = 612 neurons total, mean ± s.d.). eYFP expression on the correct side of the brainstem was verified for all mice, and % of population eYFP+ was calculated for the unilateral (injected) Amb. When injected into *Calb1*^*Cre*^ mice, ACP (calbindin+) neurons were specifically labeled. When injected into *Ghsr*^*cre*^ mice, ACV (BChE+) neurons were specifically labeled, though note lower efficiency than the ACP neuron strategy. **e**, Immunostaining of Rostral Amb ACP neurons in *Calb1*^*cre*^ mice injected with AAV-DIO-bReaChES-eYFP vector. Two calbindin+ neurons were eYFP-positive (arrowheads), indicating bReaChES-eYFP expression. **f**, Immunostaining of caudal Amb ACV neurons in *Ghsr*^*cre*^ mice injected with AAV-DIO-bReaChES-eYFP vector. A BChE+ neuron was eYFP-positive (arrowhead), indicating bReaChES-eYFP expression. Bars, 20 μm. **g**, Distribution of eYFP expression after rostral Amb injection of AAV-DIO-bReaChES-eYFP in a *Calb1*^*cre*^ mouse. Note eYFP expression in the Amb external formation (AmbEx) and in overlapping Bötzinger complex (BötC) and pre-Bötzinger complex (preBötC) breathing control regions, with sparing of Amb compact formation (AmbC, esophageal motor neurons), Amb semicompact formation (AmbSc, pharyngeal motor neurons), Amb loose formation (AmbL, laryngeal motor neurons), facial motor nucleus (nVII), retrotrapezoid nucleus (RTN), and lateral reticular nucleus (LRt). Bar, 100um. **h**, Sagittal brainstem section showing lack of eYFP expression in dorsal motor nucleus of vagus (10N) and nucleus of the solitary tract (Sol) after AAV-DIO-bReaChES-eYFP injection into Amb in *Calb1*^*cre*^ mouse. Bar, 100um. **i**, Whole-mount immunostaining showing minimal eYFP expression in the nodose-jugular complex (NJC) after AAV-DIO-bReaChES-eYFP injection into Amb in *Calb1*^*cre*^ mouse. Bar, 100 μm. **j**, Distribution of eYFP expression after caudal Amb injection of AAV-DIO-bReaChES-eYFP in a *Ghsr*^*cre*^ mouse. Note eYFP expression in AmbEx, AmbL, and in overlapping BötC and preBötC, similar to *Calb1*^*cre*^ mice in panel g but with a more caudal distribution. Bar, 100um. **k**, Sagittal brainstem section showing lack of eYFP expression in 10N and Sol after AAV-DIO-bReaChES-eYFP injection into Amb in *Ghsr*^*cre*^ mouse. Bar, 100um. **l**, Whole-mount immunostaining showing lack of eYFP expression in the NJC after AAV-DIO-bReaChES-eYFP injection into Amb in *Ghsr*^*cre*^ mouse. Bar, 100 μm.

**Extended Data Figure 6.**
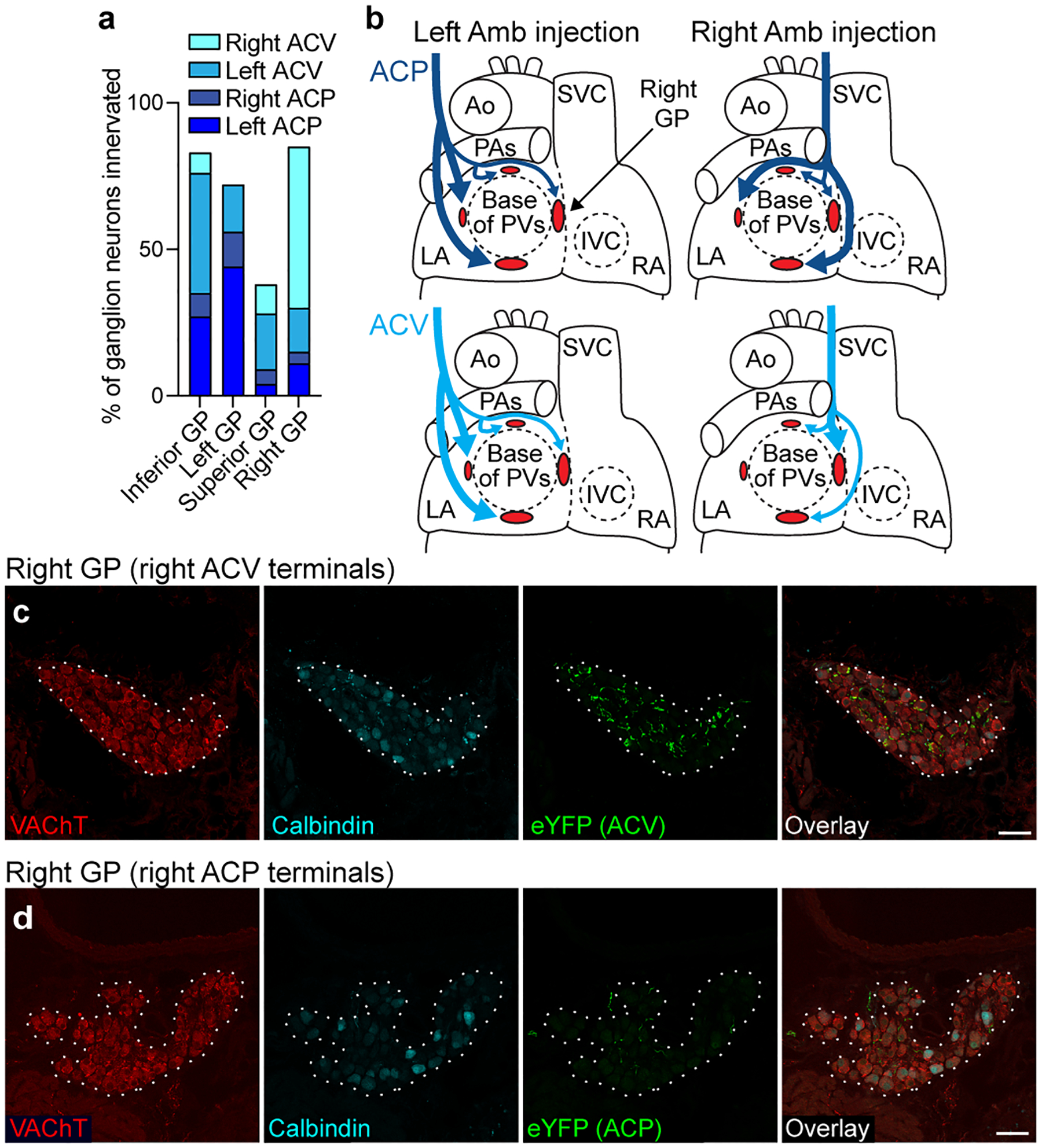
Cardiac GP projection targets of ACP and ACV neurons. **a**, Estimated proportions of ganglion neurons within each indicated ganglionated plexus (GP) that receive innervation from a given side and cell type, labeled as in Fig. 3 (*n* = 2067 neurons total, 2 mice per unilateral cell type). Remaining cells not innervated by ACP or ACV neurons are likely innervated by dorsal motor nucleus of vagus or possibly other ganglion neurons. **b**, Schematics (based on a) of left and right atria (LA and RA) of heart showing innervation of four cardiac GPs (red ovals) by left and right ACP (dark blue) and ACV (light blue) neurons. Thick arrows, dense innervation; thin arrows, sparse innervation. Note left and right ACP neurons innervate same set of GPs, whereas left and right ACV neurons innervated different sets of GPs. Ao, aorta; PA, pulmonary artery; PVs, pulmonary veins; SVC, superior vena cava; IVC, inferior vena cava. **c**, Immunostaining of the right cardiac GP (dotted outline) after right ACV fibers were labeled with eYFP. The right GP stained positive for vesicular acetylcholine transporter (VAChT), and many eYFP+ ACV fibers innervate ganglion neurons within the GP. **d**, Immunostaining of the right GP (dotted outline) after right ACP fibers were labeled with eYFP as in Fig. 3. In contrast to right ACV fibers, few eYFP+ fibers from right ACP neurons were found innervating right GP ganglion neurons. Bars, 50 μm.

**Figure 3.**
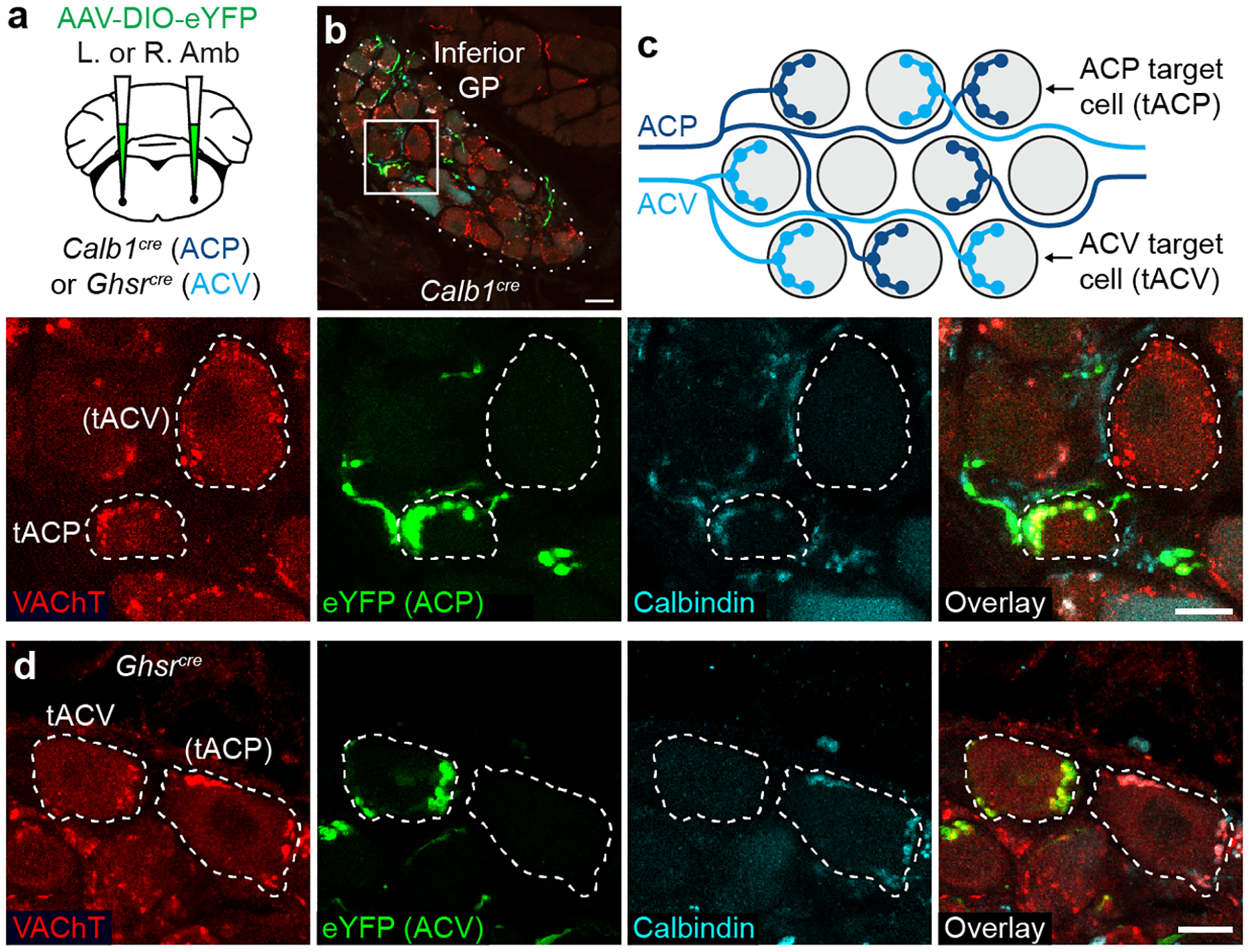
Cardiac ganglion innervation patterns of ACP and ACV neurons. **a**, Strategy for labeling ACP or ACV cardiac terminals by delivering AAV-DIO-eYFP encoding Cre-dependent eYFP reporter (green) to left (L) or right (R) Amb in *Calb1*^*cre*^ (ACP driver) or *Ghsr*^*cre*^ (ACV driver) mice. **b**, Immunostaining of ACP terminals (labeled with eYFP in *Calb1*^*cre*^ mouse) in inferior pulmonary veins GP (dotted outline). Bar, 20 μm. Inset: close-up of boxed region showing two cardiac ganglion neurons (dashed outlines), one (tACP, target of ACP) with eYFP-positive ACP input that was also Calbindin+ (ACP marker) and vesicular acetylcholine transporter (VAChT)+. An adjacent cardiac ganglion neuron ((tACV), putative target of ACV) received eYFP-, Calbindin-, VAChT+ pre-ganglionic input (red puncta), indicating it was not innervated by an ACP, but likely by an ACV neuron. Bar, 10 μm. **c**, Schematic of typical ACP and ACV innervation pattern of individual cardiac ganglion neurons (grey circles) within a GP. ACP fibers (dark blue) provide all cholinergic input for tACP neurons. ACV fibers (light blue) provide all innervation for tACV neurons, which are intermingled with tACP neurons. **d**, Immunostaining of ACV terminals (labeled as above with eYFP in a *Ghsr*^*cre*^ mouse) in two cardiac ganglion neurons (dashed outlines), one (tACV) with eYFP+, Calbindin-, VAChT+ ACV input. An adjacent cardiac ganglion neuron, (tACP), received eYFP-, Calbindin+, VAChT+ input, so was not innervated by an ACV but likely by an ACP neuron. Bar, 10 μm.

To identify the cardiac projection targets of ACP neurons, we used a genetic strategy combined with an adeno-associated virus (AAV) reporter to specifically label this cell type. The rostral Amb of *Calb1*^*Cre*^ mice was injected with a Cre-dependent AAV encoding fluorescent protein eYFP (Fig. 3a), which labeled ACP but not ACV neurons (Extended Data Fig. 5c–e), and did not drive expression in 10N or the nodose-jugular complex (Extended Data Fig. 5g–i). Three weeks after AAV injection, eYFP-positive fibers were found innervating cardiac ganglionated plexuses (GPs) on the left atrial surface surrounding the pulmonary veins (Extended Data Fig. 6a,b), the GPs known to mediate cholinergic effects on the heart^17^. When either left or right ACP neurons were labeled, most eYFP-positive fibers innervated a GP around the base of the left pulmonary veins as well as a GP around the inferior pulmonary veins (Extended Data Fig. 6a,b). Within innervated GPs, eYFP-positive fibers coursed toward specific cholinergic ganglion neurons where they terminated in cholinergic, Calbindin-positive synapses surrounding each target neuron. Fibers from a single side of the brainstem provided all the cholinergic input for a given target neuron (Fig. 3b), suggesting that ganglion neurons do not receive mixed left and right inputs. Thus, although both left and right ACP neurons generally innervate the same subset of cardiac GPs, they innervate a private subset of cholinergic ganglion neurons within these GPs.

A similar strategy was used to label ACV neurons in the left or right caudal Amb and identify their cardiac targets (Fig. 3a), substituting a *Ghsr*^*Cre*^ driver specific for ACV neurons relative to ACP neurons (Extended Data Fig. 5a–d,f,j–l). Unlike ACP neurons, ACV neurons in the right and left Amb showed different GP targeting patterns. eYFP-positive ACV fibers from left Amb innervated all GPs, whereas right ACV neurons primarily innervated a GP at the base of the right pulmonary veins (Extended Data Fig. 6a–c), a GP rarely innervated by ACP neurons (Extended Data Fig. 6a,b,d). eYFP-positive fibers of ACV neurons terminated in cholinergic, Calbindin-negative synapses that surrounded each cholinergic target neuron, providing its entire cholinergic input (Fig. 3d), just like ACP neuron targets. Strikingly, although ACP and ACV target neurons were intermingled, ACP and ACV neurons almost never innervated the same neuron (Fig. 3c,d).

We conclude that both ACP and ACV neurons innervate cholinergic cardiac GPs. Left and right ACP neurons innervate the same subset of GPs, whereas left and right ACV neurons innervate different sets of GPs. Although each GP receives a mixture of innervation from different sides and different Amb^Cardiac^ cell types, individual ganglion neurons receive all of their input from a single side and a single cell type, implying a mechanism that ensures individual ganglion neurons receive only one input. Such a mechanism could be mediated by classical axon guidance cues, repulsive cues, or cell adhesion molecules (Supplementary Table 6), and may serve to avoid interference between the two pathways.

### ACP and ACV both slow the SA and AV nodes

**Figure 4.**
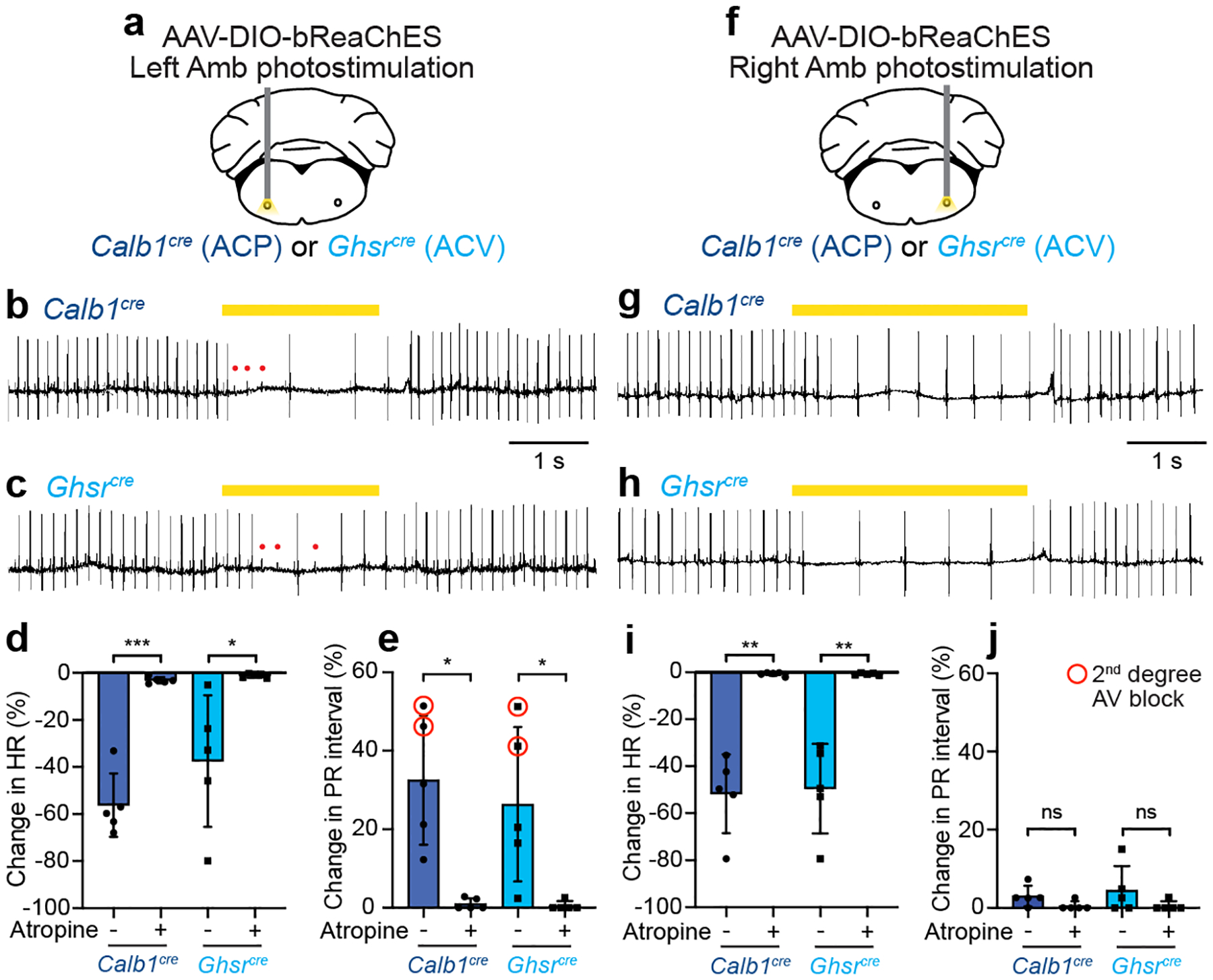
Cardiac effects of optogenetic activation of left and right ACP and ACV neurons. **a**, Strategy for optogenetic activation of left ACP or ACV neurons. AAV-DIO-bReaChES was injected into left Amb of *Calb1*^*cre*^ or *Ghsr*^*cre*^ mice to express channelrhodopsin bReaChES in ACP (*Calb1*^*cre*^) or ACV neurons (*Ghsr*^*cre*^), which were photostimulated while recording ECG. **b**, ECG trace during optogenetic stimulation of left ACP neurons in a *Calb1*^*cre*^ mouse. During stimulation (40 Hz, yellow bar), there was a rapid onset second-degree AV block (P waves (red dots) that failed to produce QRS complex (large inflection)). Also during stimulation, P-P interval was increased, indicating reduction in sinus rate. **c**, ECG trace during optogenetic stimulation of left ACV neurons in a *Ghsr*^*cre*^ mouse. Note second-degree AV block and lengthening of P-P interval, as for left ACP stimulation. **d**, Quantification of effect on heart rate (HR, from P-P interval) in b, c (*n* = 5 mice per genotype) before (−) or after (+) muscarinic antagonist atropine (*Calb1*: *p* = 0.0009, *Ghsr*, *p* = 0.04). **e**, Quantification of effect on AV node conduction in b, c. Both cell types increased P-R interval (first-degree AV block) and caused second-degree AV block in some animals (red circled data points), and effects were abolished by atropine (*Calb1*: *p* = 0.01, *Ghsr*, *p* = 0.04). **f-j**, Strategy (f) and effects (g-j) of optogenetic activation of right ACP or ACV neurons (*n* = 5 mice per genotype), as for left ACP and ACV neurons in a-e (*Calb1* HR: *p* = 0.002, *Ghsr* HR, *p* = 0.005, *Calb1* PR: *p* = 0.1, *Ghsr* PR, *p* = 0.2). Data shown as mean ± S.D. *: *p* < 0.05, **: *p* < 0.01, ***: *p* < 0.001, ns: not significant by paired two-tailed *t* test.

To determine the effects of ACP neuronal activation on the heart, we optogenetically activated this cell type in transgenic mice. Red-shifted channelrhodopsin bReaChES^18^ was expressed in ACP but not ACV neurons using a Cre-dependent AAV encoding bReaChES delivered into rostral Amb of *Calb1*^*Cre*^ mice (Extended Data Fig. 5c–e) along with a fiber optic cannula. Two to four weeks later, bReaChES-expressing ACP neurons were stimulated with a laser while recording the electrocardiogram (ECG) and respiration under isoflurane anesthesia. Photostimulation of left ACP neurons (Fig. 4a) caused an immediate, ~50% reduction in sinus rate with simultaneous prolonging of the P-R interval, occasionally leading to second-degree AV block (Fig. 4b,d,e). Upon stimulation cessation, the heart rapidly returned to normal sinus rhythm. Both SA and AV node effects of left-sided ACP stimulation were abolished by pre-treatment with muscarinic receptor antagonist atropine (Fig. 4d,e), consistent with ACP neurons mediating these effects through cholinergic ganglion neurons that activate cardiac muscarinic receptors, and not through withdrawal in sympathetic tone. Photostimulation of right ACP neurons (Fig. 4f) resulted in a similar atropine-sensitive bradycardia, but no first- or second-degree AV block (Fig. 4g,i,j). Thus, left ACP neurons slow sinus rate and AV node conduction velocity, whereas right ACP neurons slow only sinus rate.

**Extended Data Figure 7.**
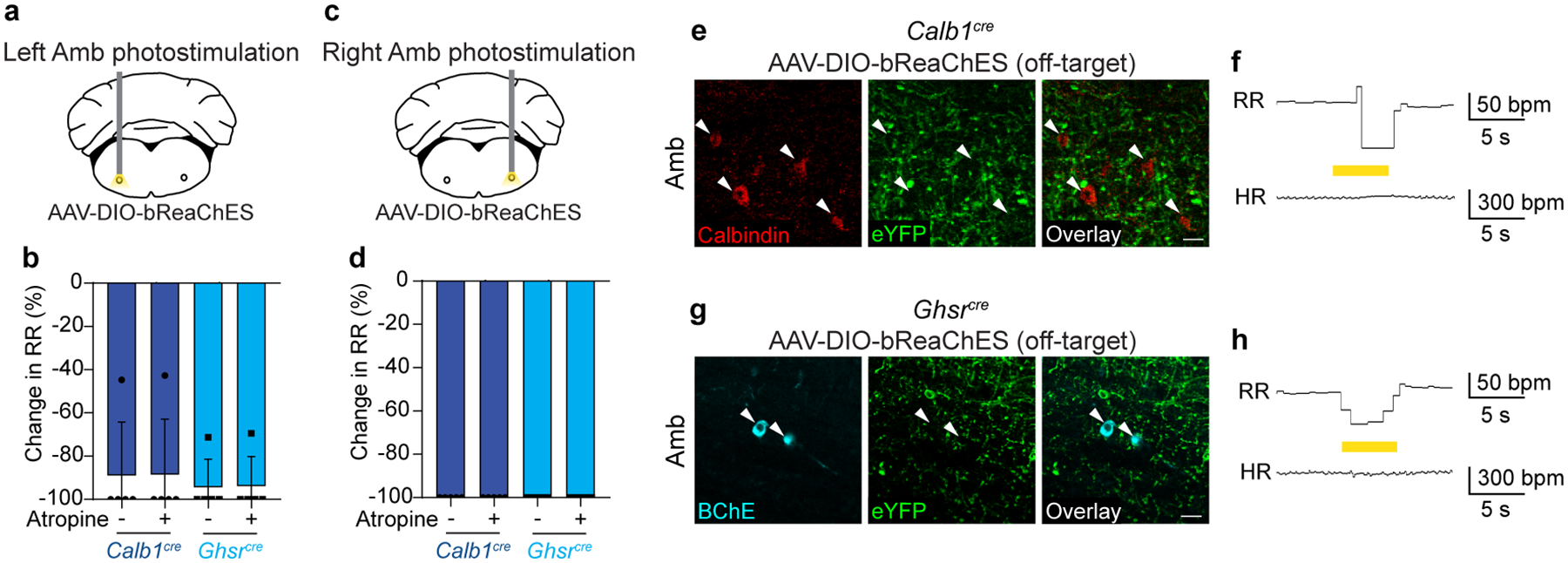
Amb optogenetic stimulation in *Calb1*^*cre*^ or *Ghsr*^*cre*^ mice results in apnea mediated by non-cholinergic neurons. Data are from same stimulation trials as Fig. 4. **a**, Schematic of optogenetic activation of left Amb ACP or ACV neurons in anesthetized *Calb1*^*cre*^ or *Ghsr*^*cre*^ mice. **b**, Before (−) atropine administration, optogenetic stimulation of left Amb *Calb1* neurons (dark blue bars) or left Amb *Ghsr* neurons (light blue bars) resulted in apnea or reduction in respiratory rate (RR) (*n* = 5 mice per genotype). After (+) atropine administration, the apnea resulting from left Amb *Calb1* or *Ghsr* neuron stimulation remained fully intact, indicating the effects are mediated by non-cholinergic neurons. **c**, Schematic of optogenetic activation of right Amb ACP or ACV neurons in anesthetized *Calb1*^*cre*^ or *Ghsr*^*cre*^ Mice. **d**, Before atropine administration, optogenetic stimulation of right Amb *Calb1* neurons (dark blue bars) or right Amb *Ghsr* neurons (light blue bars) resulted in apnea (*n* = 5 mice per genotype). After atropine administration, the apnea resulting from right Amb *Calb1* or *Ghsr* neuron stimulation remained fully intact, indicating the effects are mediated by non-cholinergic neurons. These respiratory effects are likely mediated by opsin expression in non-cholinergic interneurons of the pre-Bӧtzinger complex, an important breathing control region which overlaps significantly with Amb, contains *Calb1*- and *Ghsr*-expressing interneurons^15,52^, and where optogenetic stimulation of interneurons is known to result in apnea^53^. **e**, Immunostaining of ACP neurons in Amb in a *Calb1*^*cre*^ mouse injected with AAV-DIO-bReaChES-eYFP in Amb where injection failed to target ACP neurons (red), but still targeted nearby interneurons and fibers (green). Note lack of eYFP expression in ACP cell bodies (arrowheads). Bar, 25 μm. **f**, Respiratory rate (RR) and heart rate (HR) during optogenetic stimulation of interneurons surrounding ACP neurons in *Calb1*^*cre*^ mouse from panel e. Note decrease in respiratory rate with optogenetic stimulation of interneurons (yellow bar, 40 Hz), with no changes in heart rate. bpm, breaths per minute (for RR) or beats per minute (for HR). **g**, Immunostaining of ACV neurons in Amb in *Ghsr*^*cre*^ mouse injected with AAV-DIO-bReaChES-eYFP in Amb where injection failed to target ACV neurons (cyan), but still targeted nearby interneurons and fibers (green). Note lack of eYFP expression in ACV cell bodies (arrowheads). Bar, 25 μm. **h**, Respiratory rate (RR) and heart rate (HR) during optogenetic stimulation (yellow bar, 40 Hz) of interneurons surrounding ACV neurons in *Ghsr*^*cre*^ mouse from panel g. Note decrease in respiratory rate with optogenetic stimulation of interneurons (yellow bar), with no changes in heart rate. bpm, breaths per minute (for RR) or beats per minute (for HR).

To determine the effects of ACV neuronal activation on the heart, we used the same optogenetic approach in *Ghsr*^*Cre*^ mice. Photostimulation of left ACV neurons resulted in a ~40% reduction in heart rate with simultaneous AV block (Fig. 4c,d,e). Photostimulation of right ACV neurons reduced heart rate by ~50% but with no first- or second-degree AV block (Fig. 4h–j). Cardiac effects of left and right ACV stimulation were both fully blocked by atropine, as for ACP neurons (Fig. 4d,e,i,j). Thus, the effects of ACV neuron activation on the heart were similar to ACP: left ACV stimulation slowed sinus rate and AV node conduction velocity, whereas right ACV stimulation slowed only sinus rate. Similar cardiac responses and left-right asymmetry have been observed with vagus nerve stimulation in other mammals^19^. In addition to these cardiac responses, Amb photostimulation in *Calb1*^*Cre*^ and *Ghsr*^*Cre*^ mice also resulted in apnea or reduction in respiratory rate, however this respiratory effect was not altered by atropine (Extended Data Fig. 7a–d) and thus was attributed as an artifact of opsin expression in interneurons of the overlapping pre-Bӧtzinger complex breathing control region (Extended Data Fig. 7e–h).

We conclude that, despite their molecular and anatomical differences, optogenetic activation of ACP or ACV neurons results in virtually identical inhibitory effects on cardiac SA and AV node function, and because the effects are blocked by atropine they are likely mediated through their projections to cholinergic ganglion neurons.

### The baroreflex selectively activates ACV

**Extended Data Figure 8.**
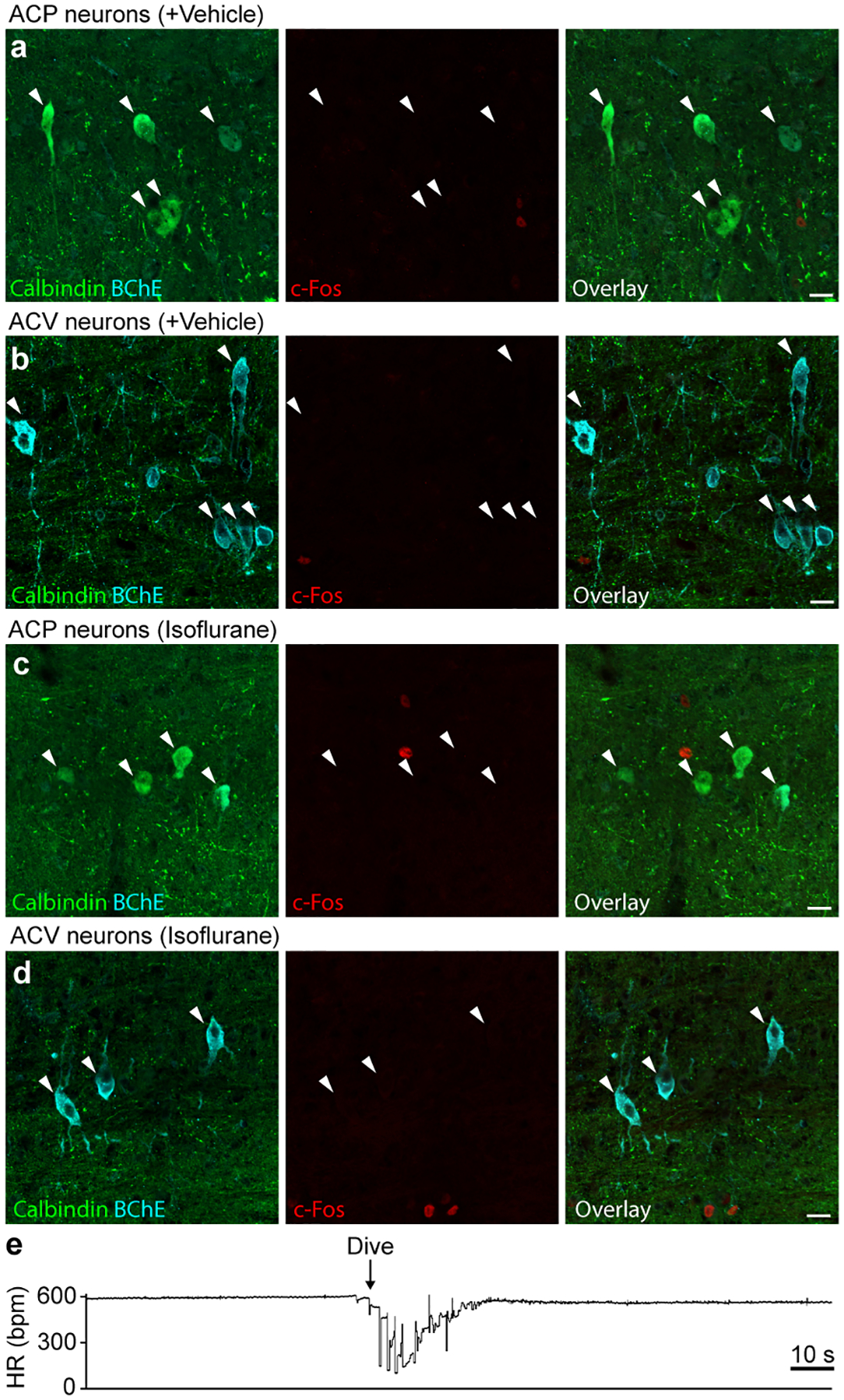
c-Fos negative control studies and heart rate response to dive reflex. **a**, Immunostaining of ACP neurons in rostral Amb following vehicle injection (see Fig. 5). Note ACP neurons (calbindin+, white arrowheads) are c-Fos negative. **b**, Immunostaining of ACV neurons in caudal Amb following vehicle injection. Note ACV neurons (BChE+, white arrowheads) are c-Fos negative. **c**, Immunostaining of ACP neurons in rostral Amb following isoflurane anesthesia without nasal immersion. Note ACP neurons (calbindin+, white arrowheads) are c-Fos negative. **d**, Immunostaining of ACV neurons in caudal Amb following isoflurane anesthesia without nasal immersion. Note ACV neurons (BChE+, white arrowheads) are c-Fos negative. Bars, 20 μm. **e**, Example heart rate trace recorded by ECG during dive reflex activation for Fig. 5 experiments. Isoflurane-anesthetized mouse underwent nasal immersion (arrow, start of dive) for 10 s. Bradycardia and AV block were observed during nasal immersion, and heart rate returned to baseline following cessation of immersion.

**Extended Data Figure 9.**
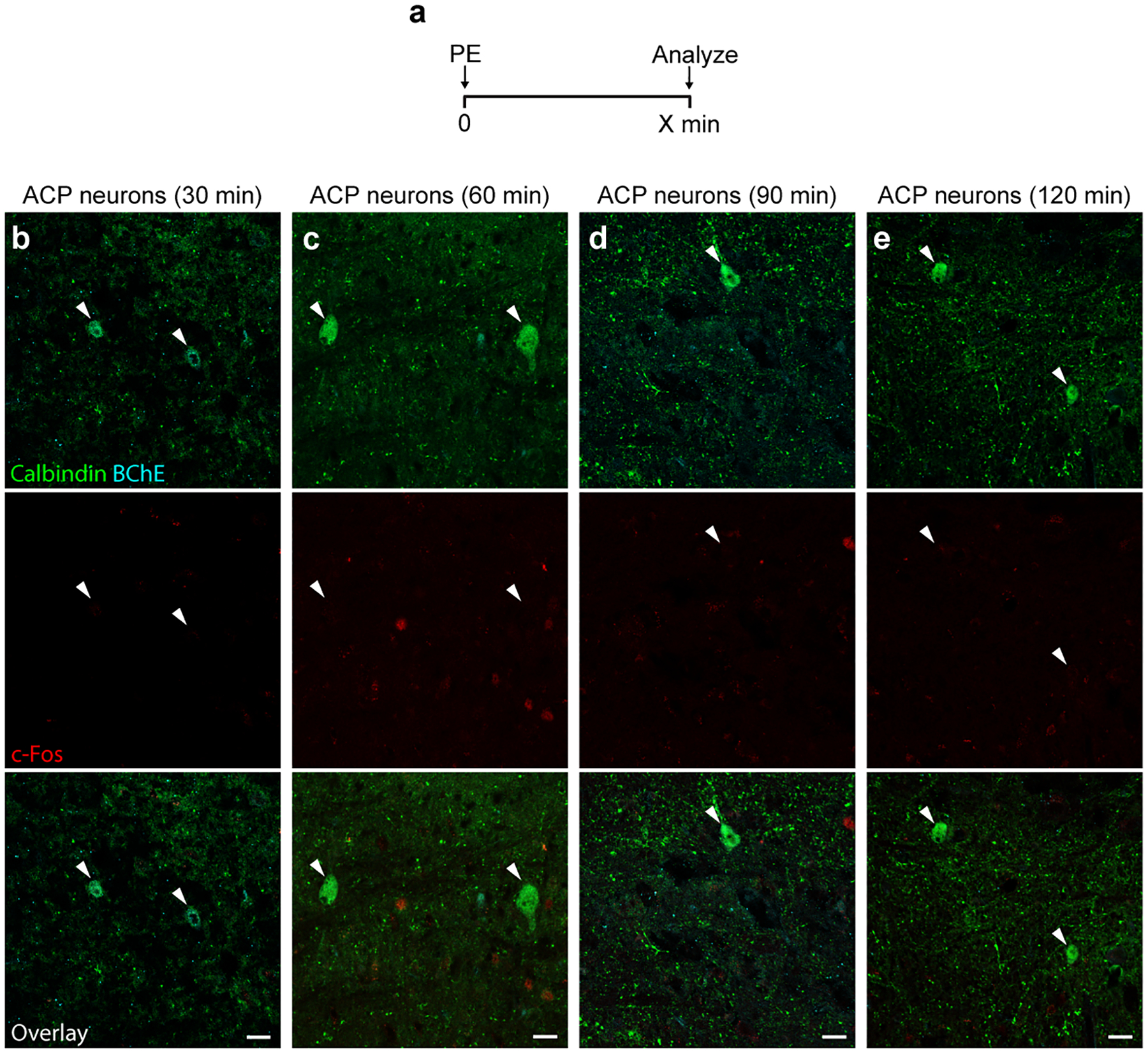
ACP neurons are not activated early after phenylephrine injection. **a**, Experimental time course paradigm. Phenylephrine (PE) (10 mg/kg, IP) was injected into awake mice and mice were sacrificed to perform immunostaining for c-Fos and the indicated Amb^Cardiac^ markers at the indicated time points following PE injection. **b-e**, ACP neurons (calbindin+, arrowheads) did not express c-Fos (red) at any of the time points (30 – 120 minutes) following PE injection. Bars, 20 μm.

**Figure 5.**
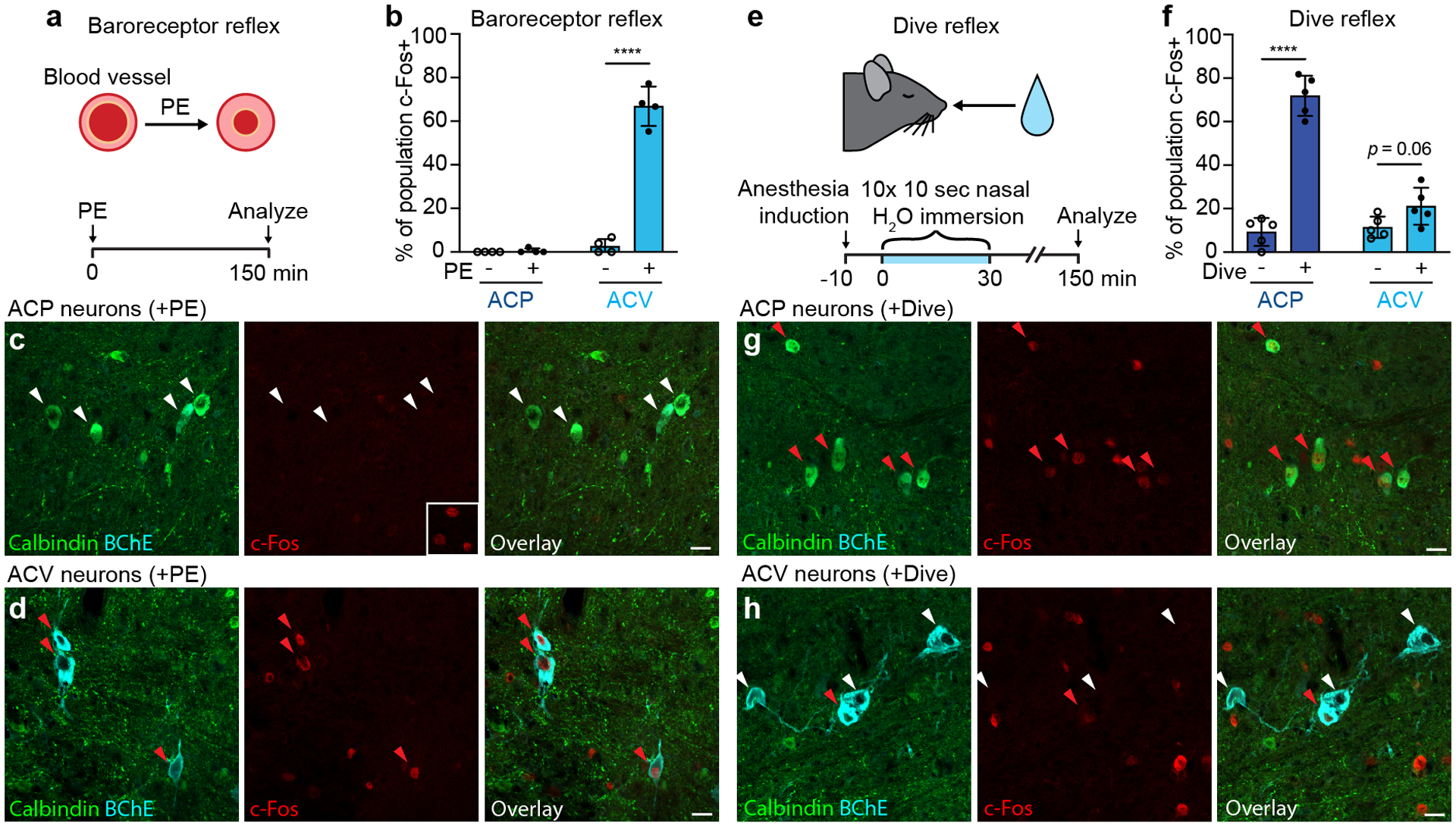
Distinct ACP and ACV activation patterns in baroreceptor and dive reflexes. **a**, Experimental design for baroreceptor reflex induction. Awake wild-type mice were administered α_1_ receptor agonist phenylephrine (PE), which causes peripheral blood vessel vasoconstriction, activating the baroreceptor reflex. After 150 minutes to allow c-Fos expression, mice were euthanized and activity of ACP and ACV neurons analyzed by c-Fos immunostaining. **b**, Fraction of ACP or ACV neurons positive for c-Fos following vehicle (- PE) or phenylephrine (+ PE) injections (*n* = 4 mice per condition, 418 total scored neurons). ACP: *p* = 0.4, ACV: *p* < 0.0001. **c**, Immunostaining for c-Fos in ACP neurons (Calbindin+, white arrowheads) in rostral Amb following baroreflex induction as above. Middle panel inset, positive immunostaining control showing c-Fos+ neurons in pre-Bӧtzinger complex of same brain section. **d**, Immunostaining for c-Fos in ACV neurons (BChE+, red arrowheads) in caudal Amb following baroreflex induction. **e**, Experimental design for dive reflex induction. Wild-type mice anesthetized with isoflurane received 10 nasal immersions (5–10 sec each) in thermoneutral water over 30 minutes, with ECG recorded to confirm dive reflex activation. Control mice were similarly anesthetized but did not receive immersions. ACP and ACV neurons were immunostained for c-Fos as in a-d. **f**, Fraction of ACP or ACV neurons that stained positive for c-Fos without (- Dive) or with dive reflex activation (+ Dive) (*n* = 5 mice per condition, 485 total scored neurons). ACP: *p* < 0.0001, ACV: *p* = 0.06. Note robust activation of ACP neurons but limited activation of ACV neurons. **g**, Immunostaining for c-Fos in ACP neurons (red arrowheads) following dive reflex induction. **h**, Immunostaining for c-Fos in ACV neurons following dive reflex induction. Note most ACV neurons are c-Fos- (white arrowheads), but rare ACV neurons are c-Fos+ (red arrowhead). Data shown as mean ± S.D. ****: *p* < 0.0001 by unpaired two-tailed *t* test. Bars, 20 μm.

We sought to identify physiological stimuli that activate ACP and ACV neurons. Amb^Cardiac^ neurons are known^20^ to mediate the baroreceptor reflex, a classic cardiovascular reflex essential for homeostatic maintenance of arterial pressure and end-organ perfusion^21^. Aortic arch baroreceptors convert increases in blood pressure to an afferent neuronal signal that results in activation of Amb^Cardiac^ neurons, which counteract the increases in blood pressure by decreasing heart rate^22^. To determine if ACP and ACV neurons are activated by the baroreceptor reflex, we induced the reflex with phenylephrine, a peripherally-acting α_1_-adrenergic receptor agonist that causes vasoconstriction and reflex activation of Amb^Cardiac^ neurons leading to bradycardia^20^. Awake wild-type mice were administered phenylephrine, and 150 minutes later animals were euthanized and ACP and ACV neurons immunostained for c-Fos, a neuronal activity marker (Fig. 5a). In vehicle-treated control mice, neither ACP or ACV neurons expressed c-Fos, indicating ACP and ACV activity is off or low under baseline conditions (Fig. 5b, Extended Data Fig. 8a,b). However, in phenylephrine-treated mice, we observed robust activation of ACV neurons (Fig. 5b,d), with ~70% staining positive for c-Fos. The effect was selective for ACV because ACP neurons remained c-Fos-negative under these conditions (Fig. 5b,c, Extended Data Fig. 9). Thus, the baroreceptor reflex selectively recruits ACV neurons.

### ACP neurons also innervate and control the lung

**Figure 6.**
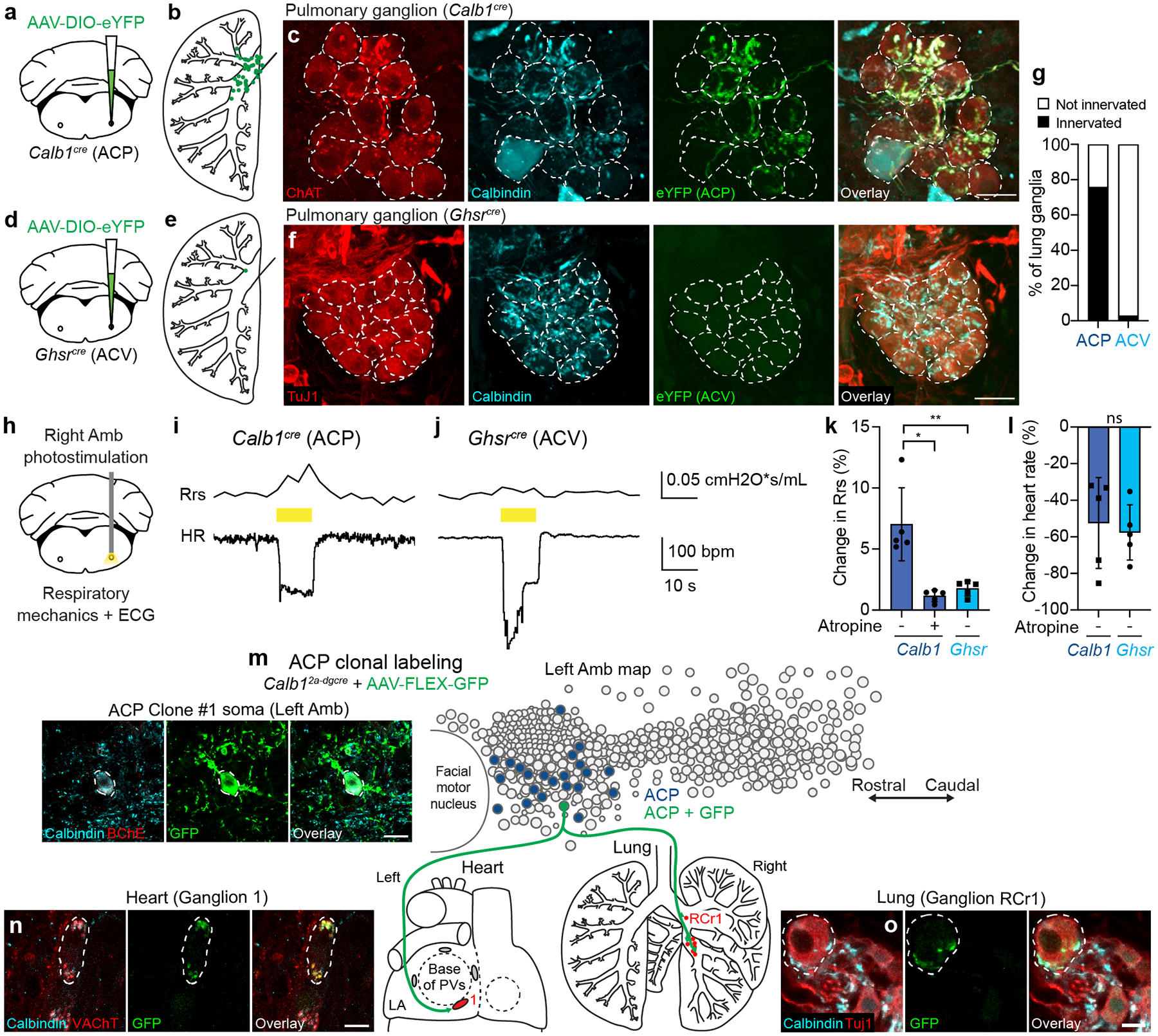
ACP neurons innervate the lung and mediate bronchoconstriction. **a**, Visualizing ACP lung terminals by injecting AAV-DIO-eYFP into Amb of *Calb1*^*cre*^ mice. **b**, Locations in lung of ACP terminals. Green dots, overlay of all innervated ganglia from 5 mice. **c**, Immunostaining of innervated cholinergic pulmonary ganglion. Ganglion neurons (dashed outlines, expressing choline acetyltransferase (ChAT)) receive cholinergic innervation (red puncta) from Calbindin+, eYFP+ ACP neurons. Bar, 25μm. **d**, Visualizing ACV lung terminals in *Ghsr*^*cre*^ mice. **e**, Locations in lung of ACV terminals. Green dots, overlay of all innervated ganglia from 5 mice. **f**, Immunostaining of representative pulmonary ganglion. Ganglion neurons (dashed outlines), expressing beta-tubulin III (TuJ1), receive Calbindin+ input (likely from ACP neurons) but no eYFP+ (ACV) input. Bar, 25μm. **g**, Percent of lung ganglia innervated by ACP or ACV neurons (*n*=33 (ACP) or 34 (ACV) ganglia, 5 mice each). **h**, Strategy for ACP or ACV optogenetic activation while measuring lung (respiratory mechanics) and heart (ECG) function. Right Amb of *Calb1*^*cre*^ or *Ghsr*^*cre*^ mice was targeted with AAV-DIO-bReaChES, then ACP or ACV neurons were activated via fiber optic. (**i**,**j**) Traces of total lung resistance (Rrs) and heart rate (HR) during optogenetic stimulation (yellow bar, 20 Hz) of ACP (i) or ACV (j) neurons. (**k**,**l**) Quantification of Rrs (k) and HR (l) changes measured simultaneously during optogenetic stimulation of ACP (*Calb1*) or ACV (*Ghsr*) neurons (*n*=5 mice each). Values are mean ±S.D. **, *p*=0.005; *, *p*=0.01; ns: not significant (*p*=0.7) by unpaired (*Calb1 vs. Ghsr*) or paired (- vs. +Atropine) two-tailed *t* test. **m**, ACP clonal labeling in *Calb1*^*2a-dgcre*^ mouse injected with limiting dose of AAV-FLEX-GFP. Left, immunostaining of soma in left Amb of the single GFP-labeled ACP neuron (Clone #1), dashed outline. Bar, 20μm. Right, location in left Amb of the GFP-labeled ACP clone (green circle). Blue circles, all other ACP neurons. Terminals of ACP clone (green fibers) were mapped in heart (left) and lung (right). and innervated parasympathetic ganglia in both organs (red ovals/circles, targeted ganglia). LA, left atrium; PVs, pulmonary veins. (**n**,**o**) Immunostaining of parasympathetic cardiac ganglion (n, Ganglion 1), showing ganglion neuron (dashed outline) receiving GFP+, Calbindin+, VAChT+ input from ACP Clone #1, and of lung parasympathetic ganglion (**o**, Ganglion RCr1), showing ganglion neuron receiving GFP+, Calbindin+ input from same clone. Bar, 10μm.

A clue to ACP neuron function came from our discovery that they also innervate another organ. The lung receives innervation from the same branch of the vagus nerve as the heart^23^, and pulmonary and cardiac function are known to be tightly coordinated^24^. In parallel studies of lung innervation, we observed Calbindin-positive innervation of pulmonary ganglia. To determine if ACP neurons project to lung, we immunostained pulmonary ganglia in *Calb1*^*Cre*^ mice after labeling ACP neurons with eYFP (Fig. 6a). Surprisingly, we found that in addition to innervating cardiac ganglia, ACP eYFP-positive/Calbindin-positive terminals also innervated most pulmonary cholinergic ganglia (75%) that surround proximal airways of the lung (Fig. 6b,g). Similar to cardiac ganglia, ACP fibers entered pulmonary ganglia and surrounded individual cholinergic ganglion neurons (Fig. 6c). In contrast with cardiac ganglia, almost all cholinergic pulmonary ganglion neurons were innervated by Calbindin-positive fibers (Fig. 6c). In similar experiments with ACV neurons, we observed only rare pulmonary ganglia (3%) that contained eYFP-positive/Calbindin-negative ACV fibers (Fig. 6d–g). Thus, ACP neurons innervate cholinergic pulmonary ganglia and are the major if not exclusive source of cholinergic input.

**Extended Data Figure 10.**
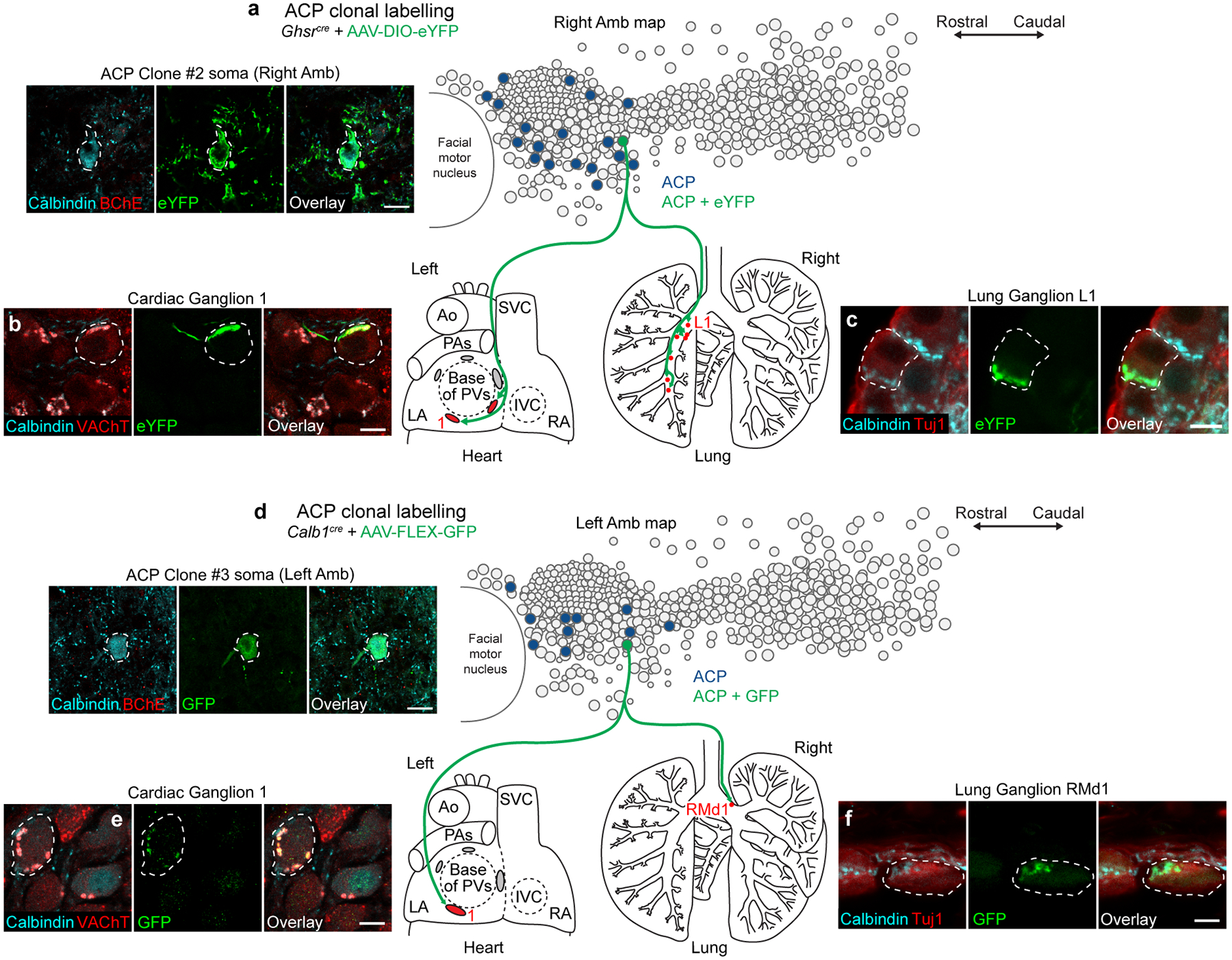
Clonal analysis of ACP neurons. **a**, ACP clonal labeling experiment in *Ghsr*^*cre*^ mouse injected with AAV-DIO-eYFP. Left, immunostaining of the soma (dashed outline) in the right Amb of the single eYFP-labeled ACP neuron in this mouse (Clone #2); note it co-stained positive for calbindin (cyan) and negative for BChE (red), confirming ACP identity. Bar, 20 μm. Right, map of ACP neurons (dark blue fill circles) in right Amb (overlay of all sagittal sections of right Amb) showing location of the eYFP-labeled ACP clone (green fill circle). Terminals of the ACP clone (green fibers) were mapped in the heart (left) and lung (right), where it was found to innervate parasympathetic ganglia in both organs (red fill ovals/circles, targeted ganglia). LA, left atrium; RA, right atrium; Ao, aorta; PA, pulmonary artery; PVs, pulmonary veins; SVC, superior vena cava. **b**, Immunostaining of parasympathetic cardiac ganglion (Ganglion 1) showing a ganglion neuron (dashed outline) innervated by ACP Clone #2. Note innervated neuron stained positive for cholinergic marker VAChT (red) and receives innervation from a cholinergic (red), calbindin-positive (cyan) fiber labeled with eYFP (green). Bar, 10 μm. **c**, Immunostaining of lung parasympathetic ganglion (Ganglion L1) showing a ganglion neuron (dashed outline) innervated by ACP Clone #2. Note innervated neuron stained positive for neuronal marker Tuj1 (red) and receives innervation from a calbindin-positive (cyan), eYFP-positive (green) fiber. Bar, 10 μm. **d**, ACP clonal labeling experiment in *Calb1*^*cre*^ mouse injected with limiting dose of AAV-FLEX-GFP. Left, immunostaining of the soma (dashed outline) in the L Amb of the single GFP-labeled ACP neuron in this mouse (Clone #3); note it co-stained positive for calbindin (cyan) and negative for BChE (red), confirming ACP identity. Bar, 20 μm. Right, map of ACP neurons (dark blue fill circles) in left Amb showing location of the GFP-labeled ACP clone (green fill circle, Clone #3). Terminals of the ACP clone (green fibers) were mapped in the heart (left) and lung (right), where it was found to innervate parasympathetic ganglia in both organs (red fill ovals/circles, targeted ganglia). **e**, Immunostaining of parasympathetic cardiac ganglion (Ganglion 1) showing a ganglion neuron (dashed outline) innervated by ACP Clone #3. Note innervated neuron stained positive for cholinergic marker VAChT (red) and receives innervation from a cholinergic (red), calbindin-positive (cyan) fiber labeled with GFP (green). Bar, 10 μm. **f**, Immunostaining of lung parasympathetic ganglion (Ganglion RMd1) showing a ganglion neuron (dashed outline) innervated by ACP Clone #3. Note innervated neuron stained positive for neuronal marker Tuj1 (red) and receives innervation from a calbindin-positive (cyan), GFP-positive (green) fiber. Bar, 10 μm.

To determine if single ACP neurons project to both heart and lung, we used sparse labeling approaches to clonally label ACP neurons in individual mice. Multiple mouse lines and AAV dose optimizations were tested to lower the efficiency of genetic labeling to increase the likelihood of labeling just a single ACP neuron (Methods). The number of GFP-labeled ACP neurons was determined by immunostaining and counting each ACP neuron in every brainstem section spanning Amb. In rare cases (3 out of >50 mice), a single ACP neuron was labeled with GFP in an individual mouse (Fig. 6m–o, Extended Data Fig. 10). In in all three of these clones (two left ACP neurons, one right ACP neuron) the single, labeled ACP neuron projected to both heart and lung, terminating in cholinergic Calbindin-positive synapses that surrounded cholinergic ganglion neurons in both organs (Fig. 6m–o, Extended Data Fig. 10). In all three cases, the ACP clone innervated only the heart and contralateral lung, innervating a variable fraction (3–100%) of the neurons in each targeted cardiac or lung ganglion and totaling ~10–30 innervated ganglion neurons in the lung and ~12–16 innervated ganglion neurons in the heart (Supplementary Table 7). Thus, single ACP neurons innervate both organs, sending dual projections to heart and lung.

Cholinergic pulmonary ganglia mediate bronchoconstriction^25^, and previous studies have shown that chemical stimulation of rostral Amb with an excitatory amino acid causes bradycardia and a simultaneous ~15% increase in total lung resistance^26^, indicating bronchoconstriction. To determine if ACP neurons mediate bronchoconstriction, ACP neurons in the right Amb were optogenetically activated in isoflurane-anesthetized, mechanically-ventilated, paralyzed *Calb1*^*Cre*^ mice while respiratory mechanics and ECG were recorded (Fig. 6h). We found that immediately following optogenetic stimulation, the previously-described bradycardia (Fig. 4) was accompanied by a ~5–12% increase in total lung resistance (Fig. 6i,k). Like the cardiac effect, ACP-mediated bronchoconstriction was abrogated by atropine administration (Fig. 6k). In contrast, optogenetic activation of ACV neurons had little effect on total lung resistance (~1–2%) (Fig. 6j,k), despite driving a similar reduction (~50%) in heart rate as ACP neurons (Fig. 6l).

We conclude that ACP, but not ACV neurons innervate and control the lung in addition to the heart, and they mediate these pulmonary effects through collateral projections to cholinergic ganglion neurons along proximal airways.

### The dive reflex activates ACP neurons

To find a physiological function for ACP neurons, we considered cholinergic reflexes involving coordinated cardiopulmonary responses. The mammalian dive reflex is a powerful, evolutionarily ancient reflex that conserves vital oxygen stores while submerged underwater^27^. Upon nasal water immersion, afferent signals to the brainstem rapidly trigger apnea, bradycardia, peripheral vasoconstriction, and bronchoconstriction. Amb mediates the dive-induced bradycardia and bronchoconstriction^26–29^, the two functions we showed above that ACP neurons control. To determine if ACP neurons are activated by the dive reflex, we induced this reflex in isoflurane-anesthetized wild-type mice by exposing them to 10 brief (10 s) nasal immersions in thermoneutral (~30°C) water over 30 minutes (Fig. 5e). ECG monitoring confirmed dive reflex activation of bradycardia during nasal immersion (Extended Data Fig. 8e). ACP neuronal activity was then assessed 120 minutes later by c-Fos immunostaining, as above in the baroreflex experiments. We found robust induction of ACP neurons by nasal immersion (~70% of ACP neurons c-Fos-positive vs. ~10% in control, non-immersion mice) (Fig. 5f,g, Extended Data Fig. 8c). Only a small fraction of ACV neurons were induced under the same conditions (~20% of ACV neurons c-Fos-positive vs. ~10% in the control) (Fig. 5f,h, Extended Data Fig. 8d). Thus, opposite to the baroreceptor reflex, the dive reflex preferentially activates ACP neurons.

## Discussion

**Figure 7.**
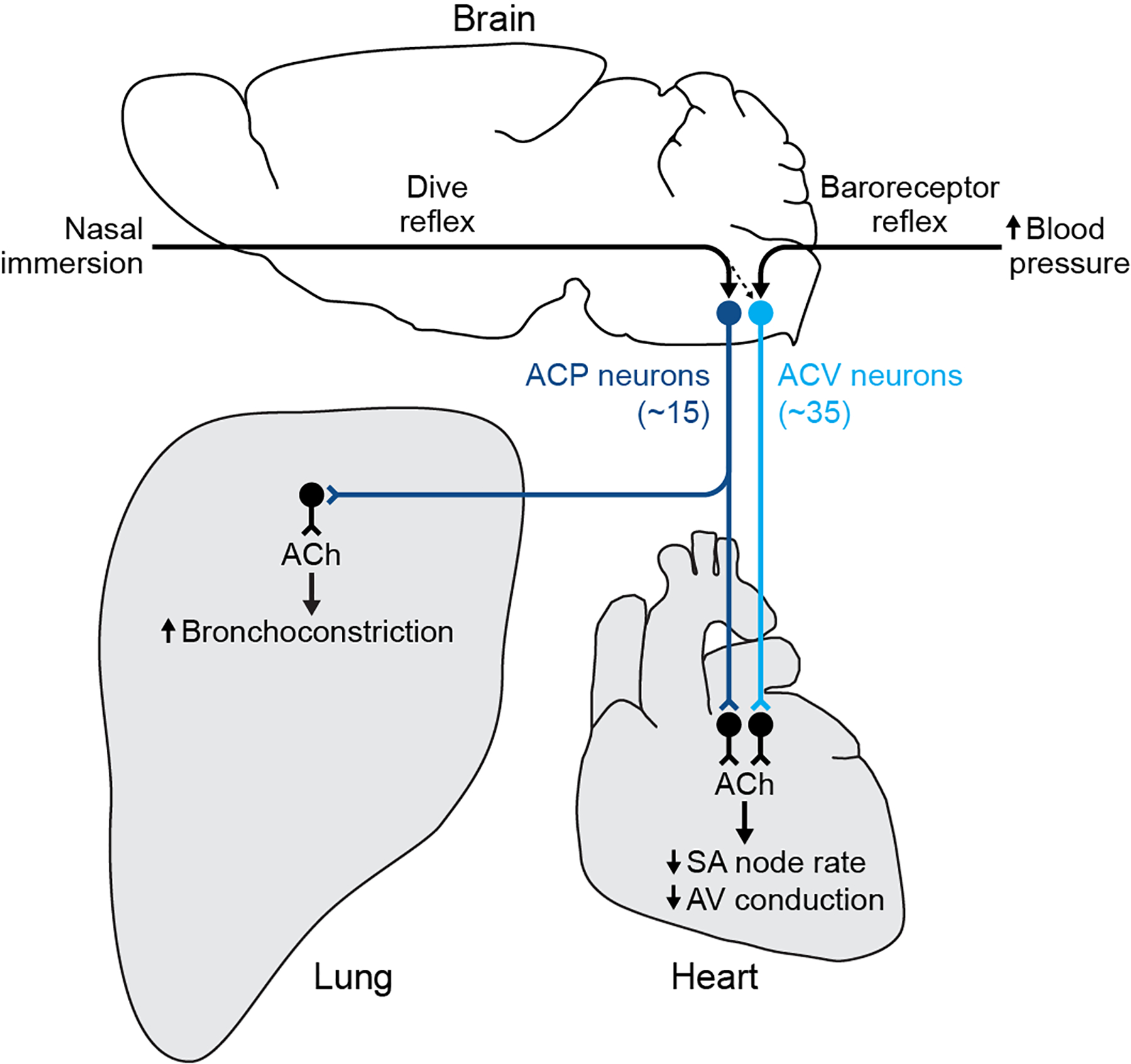
Parallel cardiovascular and cardiopulmonary control circuits. ACV neurons (light blue circles, in caudal medulla) are central to the classical cardiovascular control circuit. Increased arterial blood pressure activates aortic arch baroreceptors, initiating baroreceptor reflex that activates ACV neurons. These in turn activate cholinergic cardiac ganglion neurons (black), releasing acetylcholine (ACh) that slows SA node rate and AV node conduction velocity to homeostatically maintain blood pressure. ACP neurons (dark blue circles, in rostral medulla) are central to the newly defined cardiopulmonary control circuit. Nasal water immersion activates sensory receptors in the nose, initiating the dive reflex that activates ACP neurons. These in turn activate cholinergic cardiac ganglion neurons (black) intermingled with ACV target neurons, releasing acetylcholine (ACh) that slows SA node rate and AV node conduction velocity like ACV target neurons. Single ACP neurons also project to the lung and together they provide the dominant or exclusive input to cholinergic ganglion neurons in the lung, causing simultaneous bronchoconstriction. Low level of ACV neuron activation during dive reflex suggests some ACV neurons can be additionally recruited during dive reflex, perhaps to enhance bradycardia during colder or deeper dives. In addition to these reflex pathways, ACV and ACP likely receive input from other reflex circuits and forebrain structures (not shown), and express receptors for distinct sets of circulating hormones, to regulate cardiovascular and cardiopulmonary physiology during other states.

We have molecularly, anatomically, and functionally characterized the parasympathetic brain neurons that control cardiac function in mice, and defined two parallel circuits (Fig. 7). One is the classical cardiovascular control circuit that is activated by increases in blood pressure and mediates the baroreceptor reflex. The central neurons of this circuit are ~35 neurons in the right and left Amb (~70 total) that we designate ACV (ambiguus cardiovascular). They are selectively activated by increases in blood pressure and directly project to and activate cholinergic cardiac ganglion neurons, which slow the SA node rate and AV node conduction velocity to homeostatically maintain blood pressure. The other is a newly identified circuit that controls both cardiac and pulmonary function. It is mediated by a smaller and more rostral population of Amb neurons (~15 neurons in right and left Amb, ~30 total) we designate ACP (ambiguus cardiopulmonary). Like ACV neurons, ACP neurons project to and regulate cholinergic cardiac ganglion neurons that slow the SA and AV nodes, although through a distinct subset of cardiac ganglion neurons intermingled with those innervated by ACV. However, the most surprising and significant feature of ACP neurons is that they additionally project to and activate cholinergic pulmonary ganglion neurons that drive bronchoconstriction. Remarkably, single ACP neurons innervate both heart and lung, a rare example of a neuron that sends efferent projections to multiple organs. ACP neurons are specifically activated by the dive reflex, and thereby mediate the coordinated reduction in both cardiac and pulmonary function to conserve myocardial oxygen consumption and possibly protect the lungs while redistributing air from conducting to diffusing air spaces during water immersion.

The ACV circuit might be deployed during other states requiring a cardiac-specific parasympathetic output^4^ such as expiration (respiratory sinus arrhythmia), detection of noxious stimuli or ischemia in cardiac ventricles (Bezold-Jarisch reflex), non-REM sleep, and various emotional states. The baroreceptor reflex may be the ancestral function of the ACV circuit, given the widespread conservation of this reflex in both terrestrial and aquatic vertebrates^30^. The dive reflex is widely conserved in terrestrial vertebrates^31^, suggesting ACP neurons arose after ACV neurons, but soon after the transition to land. Indeed, this may be the ancestral function of ACP neurons because the dive reflex would have been especially valuable in early terrestrial intermediates moving between sea and land. A priority will be to identify other physiological states that engage the ACV and ACP cell types in addition to the canonical functions described here.

The identification of ACV and ACP could inform novel therapeutic approaches. While the cardiac sympathetic system can be therapeutically targeted by β-blockers with relative cardioselectivity, the cardiac parasympathetic system has resisted such targeting due to the challenges of developing muscarinic subtype-specific compounds^32^. Impaired parasympathetic signaling to the heart is independently associated with sudden cardiac death in patients with a history of myocardial infarction^7^, patients with heart failure^8^, and in otherwise healthy adults^9^. Hyperactivity or impairment in this pathway is also thought to drive baroreceptor reflex dysfunction in certain neurological disorders^21^. Thus, the molecular identification of ACV neurons and their distinct receptor profile could enable novel therapeutics that specifically target the pathological baroreceptor reflex circuit in cardiovascular and neurological disease. Another priority is to investigate whether the ACP circuit contributes to reflex bronchoconstriction^33^, pathological cardiorespiratory reflexes such as the Cushing reflex^24^, or to asthma, a disease in which airway and cardiac vagal hyperreactivity can co-occur^34^, and to target ACP neurons accordingly.

Finally, the discovery of the ACV and ACP circuits provides a cellular resolution glimpse into how the central autonomic nervous system is organized to coordinate intra-organ as well as inter-organ physiology. It is now important to employ the approach described here to molecularly and functionally identify the central parasympathetic and sympathetic neurons and circuits that control all organs, an effort that will be aided by the general parasympathetic gene signature described here. Elucidating these circuits could allow precision control of specific organs, reflexes, or combinations of organs in health and disease.

## Methods

### Mice

Wild-type mice were C57BL/6NCrl obtained from Charles River (strain 027). *Calb1-IRES2-Cre* knock-in mice^36^ and *Calb1-2A-dgCre-D* knock-in mice were obtained from The Jackson Laboratory (stock 028532 and 023531), and mice heterozygous for these alleles were used for experiments. *Ghsr-IRES-Cre* knock-in mice^37^ were generously provided by J. Zigman; although variability between mice in Cre recombination efficiency has been reported for this line^37^, we used mice homozygous for the *Ghsr*^*Cre*^ allele for all experiments to increase Cre expression and recombination efficiency, and only analyzed mice in which recombination occurred and the expected BChE+ (ACV) neurons were labeled with AAV-encoded eYFP. Male or female mice were used for all experiments. Mice were housed and bred in the animal facility at Stanford University in accordance with Institutional Animal Care and Use Committee guidance and were maintained on a 12-h light–dark cycle with food and water provided ad libitum. All mouse experiments were approved by the Stanford University Institutional Animal Care and Use Committee.

### Retrograde labeling of Amb^Cardiac^ neurons

To label Amb heart-innervating neurons for subsequent single cell RNAseq or immunostaining, cholera toxin B (CTB) conjugated to Alexa Fluor 488 or 555 (Invitrogen C22841 or C22843), which has previously been used to label Amb^Cardiac^ neurons^10^, was injected into the right atrial pericardial space of postnatal day 1 (P1) wild type mice. Mice were deeply anesthetized by hypothermia by placing them on a latex-covered ice block for 5–7 minutes. Analgesia was administered using buprenorphine (0.025 – 0.05 mg/kg, SC). Mice were maintained on the ice block and a ventral incision was made in the skin overlying the right ribcage using surgical scissors. A thoracotomy was performed between the ribs overlying the right atrium using surgical scissors. A pulled glass micropipette (Drummond 5–000-2005) filled with 1% CTB conjugate (diluted in PBS + 0.05% Fast Green dye) was inserted under the pericardium and 1 uL of solution was injected using a syringe pump (World Precision Instruments UMP3) at 10 nL/s to fill the pericardial sac, while any visible leakage of CTB out of the injection site was removed with a surgical spear. The incision was closed with a suture. Mice recovered on a 37°C heating pad until the righting reflex was regained, typically within two minutes, and then were returned to the litter.

Retrograde labeling was observed in a stereotyped distribution in the “external formation” of Amb. As noted by others for rats^10^, labeling was occasionally observed in the “compact formation” of Amb, which contains esophageal motor neurons^38^. This labeling likely results from leakage from the pericardium into the adjacent esophagus after surgery, so labeled neurons in the Amb compact formation were avoided when isolating neurons for single cell RNAseq. The ACP marker Calbindin was not expressed in the compact formation of Amb (Fig. 2c,e and Allen Brain Atlas), validating this approach.

### Retrograde labeling of Amb^Laryngeal^ neurons

CTB-488 or −555 was injected bilaterally into the cricothyroid muscles of P1 wild type mice. Mice were deeply anesthetized by hypothermia and analgesia was administered using buprenorphine (0.025 – 0.05 mg/kg, SC). A ventral incision was made in the skin overlying the larynx and the fat pads and sternohyoid muscles were dissected away to reveal the cricothyroid muscles. A fine glass micropipette filled with CTB conjugate was inserted into the muscle and 10 nL of solution was injected over 1 minute to fill each cricothyroid muscle. The skin was sealed with VetBond tissue adhesive (3M). Mice recovered on a heating pad until the righting reflex was regained and then returned to the litter. 1–3 days after injection, ~28 neurons were labeled in the Amb “external formation”, exclusively in the rostral compartment below the compact formation. This distribution in the mouse Amb is similar to the observed distribution in rats^38^.

### Isolation of Amb^Cardiac^ and Amb^Laryngeal^ neurons for single cell RNAseq

P1 wild type C57BL/6NCrl mice (n = 28 mice) were injected with 1 uL CTB-488 or −555 into the pericardial space, and 16 other mice of the same age and genotype were injected with 10 nL CTB-488 or −555 into the cricothyroid muscle. P2–4 mice were anesthetized with saturating vapors of isoflurane and rapidly decapitated. The brain was immediately placed in cold (4°C) physiological artificial cerebrospinal fluid (aCSF)^39^ containing (in mM): 124 NaCl, 3 KCl, 1.5 CaCl_2_, 1 MgSO_4_, 25 NaHCO_3_, 0.5 NaH_2_PO_4_, and 30 D-glucose, equilibrated with 95% O_2_ and 5% CO_2_ (pH=7.4). Brains were embedded in 4% low melting point agarose (Invitrogen 16520050) and 220 μm sagittal slices were cut on a vibratome (Leica Biosystems) while the aCSF bath was maintained at 4°C with continuous equilibration. Slices were transferred to an electrophysiology rig equipped with an upright fluorescent microscope (Leica) and maintained for up to 4 hours in the same aCSF solution at 22°C (neonatal medullary slices of the same region remain healthy for at least 4 hours, as measured by maintenance of a physiological breathing rhythm^40^). Borosilicate capillaries (Sutter Instrument B150-86-10) pulled to a large bore diameter (~1/4–1/3 soma diameter) were lowered onto fluorescent Amb neurons near the surface of the slice and a seal was obtained on a labeled neuron. Gentle suction was applied by syringe to aspirate the soma out of the brain slice. Immediately after extraction and while still attached to the pipette, each aspirated cell was inspected under alternating fluorescent and brightfield illumination using the 40x objective to ensure that no other cells or visible debris were attached to the neuron or the pipette. The neuron was then immediately expelled into 4 μL lysis buffer^41^, snap frozen and stored at −80°C until cDNA synthesis.

### Single cell RNAseq

cDNA synthesis was performed using the plate-based SmartSeq2 protocol^41^, with 20 cycles of amplification during the PCR step. Amplified cDNA was purified twice with 0.7x AMPure beads (Fisher A63881). cDNA quality and concentration were then assessed by capillary electrophoresis on a Fragment analyzer (AATI) before sequencing library preparation. Illumina sequencing libraries for cDNA from single cells were prepared as previously described^41^. Briefly, cDNA libraries were prepared using the Nextera XT Library Sample Preparation kit (Illumina, FC-131–1096). Nextera tagmentation DNA buffer (Illumina) and Tn5 enzyme (Illumina) were added, and the sample was incubated at 55°C for 10 minutes. The reaction was neutralized by adding “Neutralize Tagment Buffer” (Illumina) and centrifuging at room temperature at 3,220 × g for 5 minutes. Samples were then indexed via PCR by adding i5 indexing primer, i7 indexing primer, and Nextera NPM mix (Illumina). Following cDNA sequencing library preparation, wells of each library plate were pooled using a Mosquito liquid handler (TTP Labtech), then purified twice using 0.7x AMPure beads. Libraries were sequenced on a NextSeq 500 (Illumina) using 75bp paired-end sequencing.

### Single cell RNAseq data analysis

cDNA sequencing reads from the the obtained Amb^Cardiac^ (191) and Amb^Laryngeal^ (77) neurons were pruned for low nucleotide quality scores and adapter sequences using Skewer^42^ (version 0.2.2), and aligned to the mm10 genome using STAR^43^ (version 2.6.1d) in two-pass mapping mode, in which the first pass identifies novel splice junctions and the second pass aligns reads after rebuilding the genome index with the novel junctions. Neurons with less than 500,000 aligned reads or more than 1800 counts of the glial gene Apoe indicating significant glial mRNA contamination were removed from the analysis before clustering, resulting in 151 Amb^Cardiac^ neurons and 52 Amb^Laryngeal^ neurons with high quality transcriptomes (mean: 1.9 million reads and 7761 genes detected per cell) (Extended Data Fig. 2a–c).

Using Seurat v3.1.4 (https://satijalab.org/seurat/), read counts per gene were normalized across cells, scaled per 10,000 and converted to log scale using the ‘NormalizeData’ function. These values were converted to z-scores using the ‘ScaleData’ command and highly variable genes were selected with the ‘FindVariableFeatures’ function (dispersion function: LogVMR). Principal components were calculated for these selected genes using the ‘RunPCA’ command. Clusters of cells with similar expression profiles were detected using the Louvain method for community detection including only biologically meaningful principal components (see below) to construct the shared nearest neighbor map, as implemented in the ‘FindClusters’ function (resolution: 0.4). Differentially-expressed genes for each defined cluster were identified using the ‘FindMarkers’ command in Seurat using the Wilcoxon rank sum test.

Although all neurons that passed quality control expressed high levels of neuronal-specific genes (Extended Data Fig. 2d), they also contained low levels of glial transcripts (Extended Data Fig. 2e) presumably from attached glial mRNA, processes, or cells that were not visible under a 40x objective. To exclude such non-neuronal genes from the clustering, principal components containing non-neuronal genes were excluded. The 3 identified neuronal clusters had similarly low levels of glial gene expression (Extended Data Fig. 2e), indicating that these genes did not drive clustering. In addition, all differentially expressed genes between neuronal types (Supplementary Tables 1–6) examined in the Allen Brain Atlas or localized by *in situ* labeling in this paper were expressed in Amb neurons and not glia, further demonstrating that the Amb^Cardiac^, Amb^Laryngeal^, ACP, and ACV cell types were separated based on Amb neuronal genes. Although scRNAseq was performed at an early postnatal time point at which cardiac parasympathetic innervation is occurring^44^, ACP and ACV marker expression was conserved between neonatal and adult time points as assayed by immunostaining (Extended Data Fig. 4) and the Allen Brain Atlas (Supplementary Table 6); however, there may be subtle changes in gene expression not detected by these methods.

### AAV cloning, injections, and fiber optic implantations

To optimize AAV transduction efficiency, we tested major serotypes that target neurons (AAV1, 6, 8, 9, DJ) and found that AAV6 gave the best transduction of Amb neurons. We also tested three different promoters (Ef1a, hSyn, CAG) and found that CAG gave the best expression in Amb neurons. The recombinant adeno-associated viral vector AAV6-CAG-DIO-bReaChES-TS-eYFP, used for projection mapping experiments (denoted as AAV-DIO-eYFP) and optogenetic experiments (denoted as AAV-DIO-bReaChES), was produced by replacing the ChR2-eYFP sequence in pAAV-CAG-DIO-ChR2(H134R)-eYFP (Addgene #127090) with the sequence for bReaChES-TS-eYFP, a red-shifted channelrhodopsin^18^, and virions were produced at the Janelia Viral Tools facility (3.21×10^12^ viral genomes per mL). The same AAV-injected mice used for optogenetic experiments (Figs. 4, 6) were subsequently used for projection mapping (Figs. 3, 6) by visualizing the eYFP-tagged neuronal terminals as detailed below. One *Calb1*^*Cre*^ mouse injected into Amb with AAV6-CAG-Flex-GFP (UNC Vector Core, 3×10^12^ viral genomes per mL) was included in the Fig. 6 ACP lung projection mapping experiments.

Adult mice (> 6 weeks of age) were anesthetized with 3% isoflurane (for induction, and 1–2% for maintenance) for AAV injections. Anesthetized mice were placed in a stereotactic instrument (David Kopf Instruments, Model 940), with body temperature maintained at 37°C using a feedback-controlled heating pad (Physitemp, TCAT-2LV). Mice were pre-treated with analgesic (carprofen 5 mg/kg SC and buprenorphine SR 0.5–1.0 mg/kg SC). To target ACP neurons in the rostral Amb, 400 nL AAV vector was injected into *Calb1*^*Cre*^ mice at the following stereotactic coordinates: 2.4 mm caudal to lambda, ±1.33 mm lateral to lambda, 4.9 mm ventral to the brain surface. To target ACV neurons in the caudal Amb, 500 nL AAV vector was injected into *Ghsr*^*Cre*^ mice at 2.95 mm caudal to lambda, ±1.3 mm lateral to lambda, 4.8 mm ventral to the brain surface. Immediately following AAV injection, a fiber optic cannula (Thorlabs CFM12L05–10) was implanted 350 μm (ACP) or 500 μm (ACV) above the injection site and secured to the skull with dental cement (Parkell C&B Metabond). Mice in which the AAV transgene was not expressed in Amb neurons were excluded from analysis (with the exception of Extended Data Fig. 7e–h where we specifically characterized the non-Amb neurons). Mice recovered for 2–4 weeks before optogenetic and projection mapping experiments.

### Optogenetic stimulation with ECG

Mice injected with AAV6-CAG-DIO-bReaChES-TS-eYFP and recovered as above were anesthetized with isoflurane (3% induction, 1–2% maintenance) and body temperature maintained at 37°C. Single lead ECG was recorded using needle electrodes (ADInstruments MLA1213), an ADInstruments Octal Bio Amp, and an ADInstruments PowerLab data acquisition system. Respiration was simultaneously recorded using a spirometer (ADInstruments). The implanted fiber optic cannula was connected using a fiber optic cable (Thorlabs M77L01) to a 577 nm laser (CNI Laser). Laser light was delivered using the following parameters: 10–15 mW power from the fiber tip, 10 ms pulse width, 40 Hz. To assess the role of muscarinic receptors, atropine (Sigma A0132, dissolved at 50 mg/mL in ethanol, then prepared as a 0.5 mg/mL working solution in PBS) was administered (10 mg/kg, I.P.) and optogenetic stimulation was repeated 20 minutes later. To calculate the percent change in heart rate, the minimum sinus rate (60 divided by the P-P interval in seconds) during stimulation was subtracted from the sinus rate immediately before stimulation and divided by the pre-stimulation sinus rate. To calculate the percent change in P-R interval, the P-R interval immediately before stimulation was subtracted from the maximum P-R interval during stimulation and divided by the pre-stimulation P-R interval.

### Optogenetic stimulation with respiratory mechanics and ECG

Mice injected with AAV6-CAG-DIO-bReaChES-TS-eYFP and recovered for 3–4 weeks as above were anesthetized with 3% isoflurane, tracheostomized with an 18G cannula, and attached to the Flexivent (SCIREQ) as previously described^45^. Core temperature was maintained at 36–37°C with a heating pad, with the Flexivent delivering 1–2% isoflurane in pure oxygen. The mice were ventilated at 10 mL/kg at 150 breaths/minute with a positive end-expiratory pressure (PEEP) set at 3 cm H2O. ECG was recorded as described above. Prior to the evaluation of respiratory mechanics, mice were paralyzed with vecuronium bromide (Sigma 76904, 0.1–0.2 mg/kg i.p.) to stabilize the airways and eliminate breathing efforts. Vecuronium was used due to its lack of vagolytic properties. The compliance, elastance, and overall resistance of the respiratory system (Rrs) were measured every 3 seconds with the Snapshot-150 maneuver before, after, and during optogenetic stimulation (10 s, 20 Hz), with the entire trial lasting ~50 seconds. The trial was repeated 20 minutes after atropine (10 mg/kg, I.P.) delivery. To calculate the relative change in Rrs, the difference between the maximum Rrs value obtained during optogenetic stimulation and the average baseline Rrs was divided by the average baseline Rrs, which was defined as the average of the Rrs values measured in the 10 seconds prior to optogenetic stimulation.

### Induction of baroreceptor reflex and dive reflex

Wild-type juvenile (postnatal day 21) mice were used for all baroreceptor and dive reflex experiments because the dive reflex is more pronounced earlier in life^27^. To induce the baroreceptor reflex, awake mice in their home cage were either injected with phenylephrine (10 mg/kg, I.P.) (Tocris 2838, dissolved in PBS) to induce peripheral vasoconstriction, or with PBS vehicle as control. Mice remained in their home cages and were transcardially perfused 150 minutes following injection, to allow time for pressor effects and baroreflex activation (~20 min)^46^ and c-Fos protein expression (~120 min)^47^, and brains were then fixed and immunostained for neuronal activity markers as described below. In control studies, we found that ACP neurons were not activated by phenylephrine injection 30, 60, 90, and 120 minutes before perfusion (Extended Data Fig. 9), the same result obtained at the 150 minute time point (Fig. 5), consistent with prior studies showing rostral Amb neurons are not barosensitive^48^.

To induce the dive reflex, mice were first anesthetized with isoflurane (3% induction, 2% maintenance) and maintained on a 37°C heating pad while recording single-lead ECG, and then subjected to nasal immersion. Anesthetized mice in the Dive condition received a total of 10 nasal immersions in thermoneutral (~30°C) water (every 3 minutes over a 30-minute period). Immersions lasted between 5–10 s and were terminated when stable reflex bradycardia was observed on the ECG recording. Similar water temperature and dive immersion times have been used for dive reflex studies in awake rats^49^ and mice^50^. Isoflurane control mice were anesthetized and maintained identically without nasal immersions. All mice remained under anesthesia continuously until transcardial perfusion 120 minutes after completion of the 30 min dive period, and brains were then fixed and immunostained for neuronal activity markers as described below.

Classical neuroanatomical and ablation studies localized the key neurons controlling the baroreceptor and dive reflexes to Amb^Cardiac^ neurons^20,27^ and excluded contributions from other brainstem nuclei, consistent with our optogenetic stimulation, activity mapping, and neuroanatomical data. However, despite optimization of the AAV vector and promoter (see Methods, above, AAV cloning, injections, and fiber optic implantations) we were unable to achieve the high transduction efficiencies necessary to demonstrate a requirement for ACV and ACP in the baroreceptor and dive reflexes and formally exclude a contribution from the non-activated Amb^Cardiac^ cell type, since activation of small subsets of ACP or ACV (20–40% unilaterally) is sufficient to reproduce reflex bradycardia (Fig. 4, Extended Data Fig. 5d), whereas our AAV transduction efficiencies for ACP and ACV were 60% and 20% (Extended Data Fig. 5d).

### Brain and heart immunostaining

Mice were euthanized with CO_2_, transcardially perfused with 4% paraformaldehyde (PFA), and tissues were post-fixed in 4% PFA overnight at 4°C. Brains and hearts were cryoprotected in 30% sucrose at 4°C overnight. Cryoprotected tissue was embedded in optimal cutting temperature (OCT) compound and sectioned at 25 μm (Leica CM3050S cryostat). Sections were permeabilized in PBS + 0.3% Triton X-100, blocked for 1 h in block buffer (PBS + 0.3% Triton + 10% normal donkey serum), and incubated with primary antibodies in block buffer at 4°C overnight. Slides were washed three times, incubated in secondary antibodies in block buffer for 1 h at room temperature, washed three times and a coverslip was applied with Prolong Gold antifade reagent. Primary antibodies used in this study were: Rabbit anti-Calbindin (Swant CB38, 1:2000), Chicken anti-Calbindin (Novus Biologicals NBP2–50028, 1:1000), Goat anti-BchE (R&D Systems AF9024, 1:100), Chicken anti-GFP (Aves Labs GFP-1010, 1:1000), Goat anti-VAChT (Millipore ABN100, 1:500), Rabbit anti-c-Fos (Synaptic Systems 226 003, 1:5000). Species-specific donkey secondary antibodies conjugated to Alexa Fluor 488, 568, or 647 were obtained from Life Technologies or Jackson ImmunoResearch and used at a 1:500 dilution. Stained neurons were counted manually from z-stacks acquired on a Zeiss LSM 780 confocal microscope. The presence or absence of Calbindin was used to differentiate ACP and ACV neurons (punctate background Calbindin staining outside of ACP and ACV neurons is likely Calbindin+ fibers coursing through medulla). ACP neurons were defined histologically as Calbindin-positive cell bodies with low BChE expression (distinguishing them from Calbindin-positive interneurons in the pre-Bӧtzinger complex, which express no BChE). ACV neurons were defined as BChE-positive, Calbindin-negative cell bodies. For smFISH, sections were processed with a RNAscope Multiplex Fluorescent Assay v2 kit (Advanced Cell Diagnostics) according to manufacturer instructions with the probe Mm-Ghsr-C2 (426141-C2) and immunostaining was performed afterwards as above. For c-Fos experiments, control and stimulus samples were processed and imaged together in the same immunostaining experiment to minimize variability.

For cardiac immunostaining, due to anatomical variability in ganglia locations, ganglia were divided into ganglionated plexuses (GPs) in four quadrants to compare innervation patterns across animals. To estimate the proportion of GP neurons innervated by a given cell type (Extended Data Fig. 6a), the observed proportion of innervated cells (innervated neurons / total GP neurons) was divided by the labeling efficiency of the cell type (eYFP+ cell bodies / total cell bodies of cell type on given side). For clonal labeling analysis of ACP terminals in the heart, every fourth section of cardiac ganglia was stained, and ganglion neuron counts were multiplied by four.

### Lung immunostaining

Lungs were collected and immunostained as previously described^51^. Briefly, mouse lungs were perfused and inflated with 2% low melting point agarose (Invitrogen) before fixation in 4% paraformaldehyde (PFA)/PBS at 4°C for 6 hours or overnight. Vibratome sections (300–350 μm) of individual lobes were blocked in PBS with 3% BSA, 5% normal donkey serum, and 0.5% Triton-X and incubated at 4°C with primary and secondary antibodies for three and two nights respectively. Following staining, the sections were dehydrated in methanol and cleared with benzyl alcohol:benzyl benzoate (BABB) for imaging. The following primary antibodies were used: 1:500 rabbit anti-Calbindin (Swant CB38a), 1:100 goat anti-ChAT (Millipore AB144P), 1:500 chicken anti-GFP (Aves GFP-1010), and 1:300 mouse anti-Tuj1 (Covance MMS-435P or R&D MAB1195). All Alexa-Fluor-conjugated, species-specific secondary antibodies from Jackson ImmunoResearch or Invitrogen were used at 1:250. Ganglia in extrapulmonary airways were not examined.

### Clonal labeling of ACP neurons

For clonal labeling of ACP neurons, multiple AAV-based approaches were tested in parallel to reduce the efficiency of ACP neuron labeling and increase the likelihood of labeling just a single ACP neuron. To determine whether a single ACP neuron had been labeled, one month after AAV injection sagittal sections (25 μm) of the entire Amb including >150 μm of laterally surrounding tissue were immunostained for GFP and robust ACP and ACV markers (Calbindin and BChE). Each tissue section (25 μm, ~15 sections per Amb) was optically sectioned (~4 μm) and systematically scanned for labeled ACP cell bodies (typical diameter: ~20 μm). We excluded any mouse (n = 10 mice excluded) in which any brainstem section contained a fold, damage, or inadequate staining, because a labeled cell in the affected area could have been missed. All ACP neurons were counted by examining each optical section from all tissue sections, and only mice in which all Amb sections were intact (n = 48 mice) and only a single ACP neuron was labeled with GFP (n = 3 of 48 mice) were included in the analysis. For Clone #1 (Fig. 6m–o), 200 nL of AAV6-CAG-FLEX-ArchT-GFP (UNC Vector Core, 5×10^12^ viral genomes per mL) (denoted AAV-FLEX-GFP) was injected into the rostral Amb of *Calb1*^*2a-dgCre*^ mice, which were then injected with 200 mg/kg trimethoprim (IP) five days later and sacrificed one month after AAV injection. Of the 21 injected mice, only one contained a single labeled ACP neuron (in left Amb). Clone #2 (Extended Data Fig. 10a–c) was identified in a rare case in which injection of AAV6-CAG-DIO-bReaChES-TS-eYFP (denoted AAV-DIO-eYFP) in a *Ghsr*^*Cre*^ mouse labeled a single ACP neuron (in right Amb). For Clone #3 (Extended Data Fig. 10d–f), 50 nL of AAV6-CAG-FLEX-ArchT-GFP was injected into the rostral Amb of *Calb1*^*Cre*^ mice. Of the 37 injected mice, only one contained a single labeled ACP neuron (in left Amb).

### Statistics and data collection

All results are presented as mean ± S.D. with all data points displayed. All statistical analyses were performed with GraphPad Prism. All statistical tests used are listed at the end of each figure legend. Statistical significance was set at *p* < 0.05. No statistical methods were used to predetermine sample size. Mice were randomized to reflex groups and then randomized to control or stimulus conditions. No blinding was used for data collection. All physiological recordings were analyzed with the same automated scripts applied to all mice.

## Extended Data

## Supplementary Material

Supplementary Table 1

Supplementary Table 2

Supplementary Table 3

Supplementary Table 4

Supplementary Table 5

Supplementary Table 6

Supplementary Table 7

Supplementary Table Legends

Source Data ED Fig 2

Source Data ED Fig 4

Source Data ED Fig 5

Source Data ED Fig 6

Source Data ED Fig 7

Source Data Fig 2

Source Data Fig 4

Source Data Fig 5

Source Data Fig 6

## Data Availability

Singe cell RNA sequencing data from Amb^Cardiac^ and Amb^Laryngeal^ neurons is available on the Gene Expression Omnibus (GEO accession number GSE198709).
