# Supplementary material for: Molecularly defined circuits for cardiovascular and cardiopulmonary control
Source: Nature. Author manuscript; Available in PMC 2022 Jul 20. (PMC9297035; doi:10.1038/s41586-022-04760-8)
Supplement: Supplementary Table 3 [file NIHMS1818629-supplement-Supplementary_Table_3.pdf]

Supplementary Table 3. Selected genes enriched in Amb^{Cardiac} or Amb^{Laryngeal} neurons arranged by function

Known or predicted function	Amb^{Cardiac}	Amb^{Laryngeal}
Transcription factor	*Tbx3*[1], *Zcchc12*[1], *Zfhx4*[2]	*Lhx4*, *Phox2a*, *Rorc*, *Pou3f1*, *Esrrg*
Regulator of RNA splicing	*Celf4*[1], *Celf6*[1]	
Nuclear export factor	*Hcfc1r1*[2]	
Fast motor neuron fate specification		*Dlk1*
Axon guidance	*Efna5*[2], *Sema5a*[1]	*Gfra1*, *Sema5b*, *Epha3*, *Ephb1*
Neurotrophic factor	*Nenf*[1]	
Neurotrophic factor receptor	*Gfra2*[4]	
Cell adhesion	*Cdh8*[1], *Cntn5*[1], *Cbln4*[4], *Nrxn3*[1]	*Tmem132c*, *Pcdh8*, *Adgrg2*
Phospholipid synthesis	*Agpat4*[1]	
Glycosylation		*Galnt16*, *Fut10*, *B3galt5*
Sialylation		*St3gal1*
Cytoskeleton regulation	*Stmn1*[1]	
Motor protein	*Myo1b*[2]	
Extracellular matrix regulation		*Timp3*, *Pmp22*, *Col24a1*, *Hapln4*
Ion channel	*Kcna5*[1]	*Kcna1*, *Itpr3*, *Trpm3*
Neuropeptide	*Scg2*[1]	*Calca*, *Calcb*, *Pnoc*, *Uts2*, *Trh*, *Cartpt*
Neuropeptide receptor	*Npy2r*[2]	*Ackr3*, *Tacr1*
Neurotransmitter receptor	*Cnr1*[1], *Grm5*[3], *Gabrb1*[1]	*Kctd12*, *Adra1d*, *Gria3*
Neurotransmitter degrading enzyme	*Maoa*[2]	
Synaptic vesicle release	*Cplx2*[1], *Syt5*[1], *Stx1a*[1], *Vamp2*[1]	*Syt12*
Dendritic spine maintenance	*Dgkb*[1]	*Dgkk*
lncRNA	*AW549542*	
Other signaling	*Pgrmc1*[1], *Rgs4*[4], *Fgf13*[1], *Met*[2], *S100a11*[2]	*Plch1*, *Itpr3*, *Lgr5*, *Htra1*, *Prss12*, *Ptchd4*, *Tesc*, *Ak5*, *Bag3*, *Hipk4*
Unknown function	*Snca*[1], *Tm4sf4*[2], *A730017C20Rik*[1], *Tmem47*[1], *Tmem178*[1]	*Slc44a5*, *Rhbdl3*, *Hhatl*, *A830018L16Rik*, *Plekhd1*

[1]Amb^{Cardiac}-specific gene was also expressed in both the dorsal motor nucleus of vagus and lacrimal/salivatory nucleus
[2]Amb^{Cardiac}-specific gene was also expressed in the dorsal motor nucleus of vagus but not the lacrimal/salivatory nucleus
[3]Amb^{Cardiac}-specific gene was also expressed in the lacrimal/salivatory nucleus but not the dorsal motor nucleus of vagus
[4]Amb^{Cardiac}-specific gene was not expressed in the dorsal motor nucleus of vagus or the lacrimal/salivatory nucleus
