# Supplementary material for: Molecularly defined circuits for cardiovascular and cardiopulmonary control
Source: Nature. Author manuscript; Available in PMC 2022 Jul 20. (PMC9297035; doi:10.1038/s41586-022-04760-8)
Supplement: Supplementary Table 6 [file NIHMS1818629-supplement-Supplementary_Table_6.pdf]

Supplementary Table 6. Selected genes enriched[1] in ACP or ACV neurons arranged by function

Known or predicted function	ACP	ACV
Transcription factor	***Tbx2, Onecut1, Onecut3, Eya4***, *Pou4f1*	***Hoxa5, Hoxc4, Hoxb5, Hoxc5***
Neuronal development	***Tenm1,*** *Mab21l2,* ***Plxdc2***	***Adamts18***
Neurite outgrowth	***Dpy19l1***	***Cd9,*** *Socs2,* ***Dgkg***
Neuron repulsion	*Megf11*	
Axon outgrowth	*Rasgrf1*	
Axon guidance	***Sema3d,*** *Sema3c, Ptprd, Rgmb,* ***Gfra2,*** *Ntng2,* ***Dcc***	*Gfra1*
Cell adhesion	***Cntn4, Pcdh18, Cldn1, Pcdh10, Cdh2,*** *Cbln2, Cntn3,* ***Cntn6***	***Ctnna2, Cd24a,*** *Cd44*
Synapse formation	***Slitrk5***	
Motor neuron survival	*Lifr*	***Arg1***
Ion channel	***Kcnn2***, ***Kcnj12***	***Ano2***
Calcium buffer	***Calb1***	
Neuropeptide	***Uts2b***	
Neuropeptide receptor	***Trhr, Tacr3, Mc4r, Cckbr,*** *Agtr1b*	***Sstr5, Sstr2, P2ry1***
Circulating hormone receptor		***Ghsr, Rxfp2, Lepr***
Neuropeptide degrading enzyme	***Trhde***	
Neurotransmitter receptor	***Gabre***, *Gabrq*, ***Adra2a***	***Htr3b***
Nonspecific cholinesterase		***Bche***
Regulation of neurotransmitter signaling	*Tmem163,* ***Agtrap***	
Synaptic vesicle release	*Sv2b*	
Synaptic plasticity		***Akap7***
Protease	***Pappa2***	
Thyroid hormone transporter	***Slc16a2***	
Extracellular matrix	***Sgcz***	
Cytoskeletal regulation	***Avil***	
lncRNA	***Gm16551***	
Other signaling	*Thsd7a,* ***Gulp1, Csmd1, Rgs8, Bambi***	***Plcb4***, *Adcy8,* ***Igf1, Dlc1, Mfng,*** *Tspan18*
Unknown function	***D930028M14Rik, Slc35f4, Tmem47,*** *Lcp1,* ***Lancl3***, *Vwa7*	*Parm1,* ***Pacrg,*** *Cpne8,* ***Thsd7b, Susd5, Tmem178,*** *Egfem1,* ***Il17f***

[1] Boldface genes were not expressed in Amb^{Laryngeal} neurons
