# Supplementary material for: Molecularly defined circuits for cardiovascular and cardiopulmonary control
Source: Nature. Author manuscript; Available in PMC 2022 Jul 20. (PMC9297035; doi:10.1038/s41586-022-04760-8)
Supplement: Supplementary Table 7 [file NIHMS1818629-supplement-Supplementary_Table_7.pdf]

Supplementary Table 7. Projection targets of single ACP neurons

Clone ID	Method	Amb injected	Cardiac ganglion	Lung ganglion[1]	Innervated neurons[2]	Total neurons in ganglion	% of ganglion innervated
Clone #1	*Calb1*$^{2a\text{-}dgCre}$	Left	Ganglion 1		16	156	10
				RCr 1	3	109	3
				RCd 2	3	51	6
				RCd 3	2	6	33
				RCd 4	8	17	47
				RCd 5	7	25	28
				RCd 6	3	5	60
				RCd 7	3	18	16
Heart total					16	156	10
Lung total					29	231	13
Grand total					45		
Clone #2	*Ghsr*^{Cre}	Right	Ganglion 1		8	124	6
			Ganglion 2		4	160	3
				L 1	4	51	8
				L 2	4	4	100
				L 3	3	3	100
				L 4	3	10	30
				L 5	3	9	33
				L 6	4	8	50
				L 7	1	3	33
				L 8	3	3	100
Heart total					12	284	4
Lung total					25	91	27
Grand total					37		
Clone #3	*Calb1*^{Cre}	Left	Ganglion 1		12	212	6
				RMd 1	10	56	18
Heart total					12	212	6
Lung total					10	56	18
Grand total					22		

[1] Ganglia targeted by single clones are described in proximal-distal order along the airway.
[2] Targeted cells within each ganglion were defined as those surrounded by a clear, GFP+ pericellular basket and represents a minimum number of neurons contacted.
Abbreviations: L, left lobe; Rcr, right cranial lobe; RMd, right medial lobe; RCd, right caudal lobe. The extrapulmonary airways and the right accessory lobes were not examined.
