# Supplementary material for: Molecularly defined circuits for cardiovascular and cardiopulmonary control
Source: Nature. Author manuscript; Available in PMC 2022 Jul 20. (PMC9297035; doi:10.1038/s41586-022-04760-8)
Supplement: Supplementary Table Legends [file NIHMS1818629-supplement-Supplementary_Table_Legends.pdf]

Supplementary Information

Molecularly defined circuits for cardiovascular and cardiopulmonary control

Avin Veerakumar[1,2,3], Andrea R. Yung[1], Yin Liu[1], & Mark A. Krasnow[1]*

[1]Department of Biochemistry and Howard Hughes Medical Institute,
Stanford University School of Medicine, Stanford, CA USA;
[2]Department of Bioengineering, Stanford University, Stanford, CA USA;
[3]Medical Scientist Training Program, Stanford University School of Medicine,
Stanford, CA USA

Supplementary Tables

Supplementary Tables 1, 2, 4, and 5 are supplied as Excel files.

Supplementary Table 1. Genes enriched in $Amb^{Laryngeal}$ neurons (retrograde labeled from laryngeal muscle) relative to $Amb^{Cardiac}$ (retrograde labeled from heart) ($p < 0.05$, Wilcoxon rank sum test with Bonferroni correction). Rows show marker genes and columns show p values, average log fold change, percentage of a subpopulation that expresses detectable levels of a marker gene, and Bonferroni-corrected p values.

Supplementary Table 2. Genes enriched in $Amb^{Cardiac}$ neurons (retrograde labeled from heart) relative to $Amb^{Laryngeal}$ (retrograde labeled from laryngeal muscle) ($p < 0.05$, Wilcoxon rank sum test with Bonferroni correction). Rows show marker genes and columns show p values, average log fold change, percentage of a subpopulation that expresses detectable levels of a marker gene, and Bonferroni-corrected p values.

Supplementary Table 3. Selected genes differentially expressed between $Amb^{Cardiac}$ and $Amb^{Laryngeal}$ neurons (from Supplementary Tables 1 and 2) arranged by function. When multiple genes are listed in a given cell type and function, they are ordered by statistical significance. Superscripts denote whether $Amb^{Cardiac}$-specific genes were also expressed in other brainstem parasympathetic nuclei (determined, where data was available, from Allen Brain Atlas).

Supplementary Table 4. Genes enriched in ACP relative to ACV ($p < 0.05$, Wilcoxon rank sum test with Bonferroni correction). Rows show marker genes and columns show p values, average log fold change, percentage of a subpopulation that expresses detectable levels of a marker gene, and Bonferroni-corrected p values.

Supplementary Table 5. Genes enriched in ACV relative to ACP ($p < 0.05$, Wilcoxon rank sum test with Bonferroni correction). Rows show marker genes and columns show p values, average log fold change, percentage of a subpopulation that expresses detectable levels of a marker gene, and Bonferroni-corrected p values.

Supplementary Table 6. Selected genes differentially expressed between ACP and ACV neurons (from Supplementary Tables 4 and 5) arranged by function. Genes were verified to be expressed in subsets of Amb neurons in the adult Allen Mouse Brain Atlas when available. Genes in bold font were specific to either ACP or ACV and were not expressed in $Amb^{Laryngeal}$ neurons. Non-bolded genes were specific to either ACP or ACV but were also expressed in $Amb^{Laryngeal}$ neurons. When multiple genes are listed in a given cell type and function, they are ordered by statistical significance.

Supplementary Table 7. Projection targets of single ACP neurons. Three ACP clones were labeled (Fig. 6m-o, Extended Data Fig. 10) and projection targets were analyzed in parasympathetic cardiac and pulmonary ganglia. All innervated ganglia in both organs are listed as rows containing the total number of neurons innervated within the ganglion, the total number of neurons in the ganglion, and the percent of neurons innervated within the ganglion.
